# Imaging improvements reveal guttae development and posterior fibrillar layer formation in fuchs endothelial corneal dystrophy

**DOI:** 10.1038/s41598-026-44926-2

**Published:** 2026-03-26

**Authors:** Daniel B. Zander, Anne-Marie S. Kladny, Judith-Lisa Lieberum, Philip Maier, Thabo Lapp, Stefan J. Lang, Sonja Heinzelmann-Mink, Claudia Auw-Hädrich, Günther Schlunck, Katrin Wacker, Gottfried Martin

**Affiliations:** 1 Eye Center, Medical Center, Medical Faculty, University of Freiburg, University of Freiburg, Killianstr. 5, 79106 Freiburg, Germany; 2 Augenarztpraxis Prof. Dr. Wacker, Herbolzheim, Germany; 3 Department of Ophthalmology, St. Franziskus Hospital, Muenster, Germany; 4 Klinik für Augenheilkunde, Medizinische Hochschule Brandenburg, Neuruppin, Germany; 5 Eye Clinic, University Hospital Basel, Basel, Switzerland

**Keywords:** Fuchs endothelial corneal dystrophy, Descemet membrane endothelial keratoplasty (DMEK), Guttae, Hassall-Henle bodies, Posterior fibrillar layer, Polarized light, Eye diseases, Corneal diseases, Biological fluorescence, Interference microscopy, Polarization microscopy, Ageing

## Abstract

Guttae are a hallmark of Fuchs endothelial corneal dystrophy (FECD) and the disease’s progression. A posterior fibrillar layer (PFL) that covers central guttae has been previously described, but its formation and progression remain unclear. This study aims to further investigate the characteristics of guttae and the PFL in FECD. In a well-characterized prospective FECD patient cohort, a total of 43 DMEK (Descemet membrane endothelial keratoplasty) specimens were immunostained for ZO1 or COL1, flat-mounted, and analyzed using differential interference contrast (DIC), autofluorescence (excited at 480 nm), and polarized light microscopy. Guttae and PFL were quantified and correlated with clinical data. Guttae were visualized by DIC and autofluorescence imaging and classified into two types with gradual transition: peripheral, knob-like guttae and central, flat guttae. Guttae with low autofluorescence often were covered by corneal endothelial cells (CEnC). Central guttae were found to be covered by a PFL that contained COL1 and was visualized using polarized light microscopy. The PFL displayed a fibrous structure that blurred guttae in DIC illumination. Its presence correlated with clinical parameters, such as anterior scatter and post-operative corneal edema resolution providing insight into the relation of PFL and FECD severity. The combination of DIC, autofluorescence, and polarized light microscopy provides a robust method for investigating guttae and the PFL. This could facilitate preoperative assessment of corneal donor tissue and enhance postoperative clinical diagnostics by detailed detection of guttae and PFL in patients.

**Supplementary Information:**

The online version contains supplementary material available at 10.1038/s41598-026-44926-2.

## Introduction

The corneal endothelium, a single cell layer lining the inner surface of the cornea, plays a crucial role in maintaining corneal transparency by regulating stromal hydration. With advancing age, the vitality of corneal endothelial cells (CEnCs) diminishes, and in severe cases, the cell layer may disappear entirely. Fuchs endothelial corneal dystrophy (FECD), a progressive dystrophic disorder, is characterized by the pathological deposition of extracellular material by CEnCs onto Descemet’s membrane (DM). This material forms distinct circular, knob-like protrusions known as guttae. In advanced stages of FECD, CEnCs secrete additional extracellular matrix, including collagen, creating a posterior fibrillar layer (PFL) that fills the spaces between the guttae^1–3^. Both guttae and the PFL disrupt corneal optics, resulting in visual haze that impairs vision.

Beyond causing optical disruption, the loss of CEnCs compromises the cornea’s ability to pump excess fluid from the stroma into the anterior chamber, which leads to corneal edema and progressive visual deterioration. To restore visual function, patients typically undergo Descemet membrane endothelial keratoplasty (DMEK), a procedure in which the impaired DM, including guttae and the PFL, is replaced by a donor DM bearing healthy CEnCs.

On clinical slit lamp examination and under optimal conditions, guttae are visible as small, reflective dots, while the PFL appears as a faint haze. After DMEK, guttae can be visualized in surgically extracted DM using phase-contrast microscopy; however, this technique provides limited resolution, hindering detailed analysis. To overcome this limitation, we sought to identify improved imaging methods that enhance the visualization of guttae and the PFL, facilitating both research and diagnostic applications.

In this study, we examined DMs isolated from patients undergoing DMEK, adhering to previously established protocols for prospective cohort studies^4^. The DMs were flat-mounted after immunofluorescence staining for tight junction protein 1 (TJP1 or ZO1) or collagen type 1 (COL1) to enable spatial analysis of histological features and correlation with pre- and postoperative clinical data.

We found that guttae could be visualized in remarkable detail using differential interference contrast (DIC) microscopy or by assessing autofluorescence upon excitation at 480 nm. The PFL was distinctively visible using DIC or polarized light microscopy (PLM), as well as through immunostaining for COL1. Notably, the presence of a PFL correlated clinically with increased anterior scatter and more severe corneal edema, both established indicators of progressed disease in FECD. These imaging approaches not only allow the detailed observation of guttae and PFL development but also enhance disease staging by linking histological findings to clinical severity^4–7^.

## Results

### Central guttae are structurally different from peripheral guttae

**Fig. 1:**
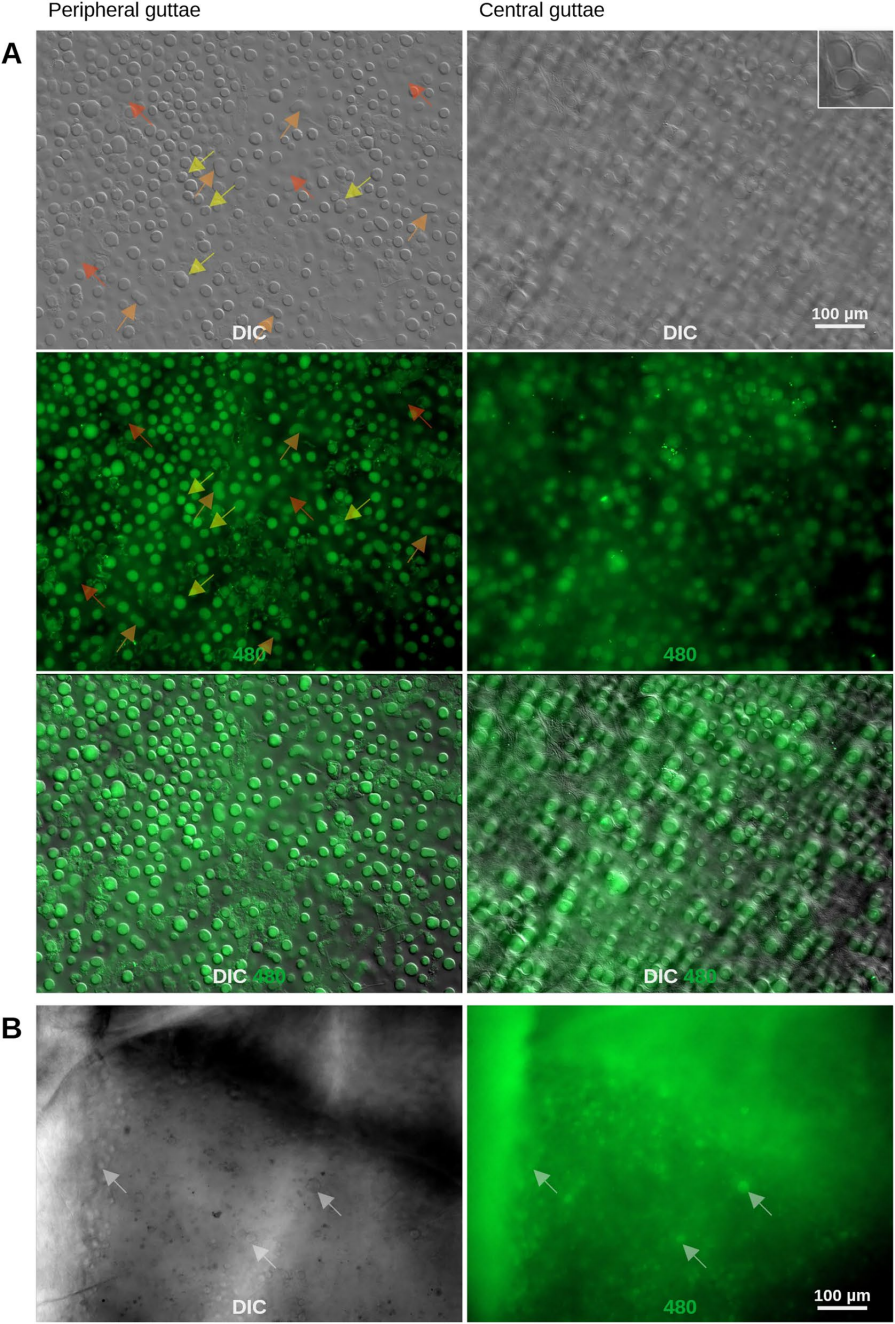
Unstained peripheral and central guttae in flat-mounted DM. (**A**) An unfixed and unstained Descemet membrane (DM) sample from a patient with Fuchs’ endothelial corneal dystrophy (FECD) flat-mounted on a slide in PBS. The multi-channel images were focussed in DIC. Peripheral guttae appear as smooth, knob-like, and clearly defined circular protrusions of varying sizes, distributed relatively evenly across the DM. Yellow arrows highlight guttae with a lobular circumference and flatter borders, deviating from the typical knob-like elevation. Orange arrows indicate guttae in different stages of unification with neighboring guttae, where original centers may still be visible. Red arrows point to guttae with minimal elevation, potentially representing early stages of formation. Central guttae differ in appearance, being flatter, more diffuse, and circular, with varying sizes and distributions. These central structures are surrounded and covered by a fine, diffuse, fibrous posterior fibrillar layer (PFL), visible as a circular structure around guttae (see inset). Sometimes, this circular structure seems to be elevated above the gutta it surrounds. Green autofluorescence was detected at 480 nm excitation, with fluorescence intensity correlating with morphological elevation as observed under DIC imaging (e.g., compare guttae marked with red arrows). (**B**) An excised and cultured whole cornea (not a flat-mounted DM) from a patient with FECD, imaged from the epithelial side. Both DIC and autofluorescence imaging revealed the presence of guttae. Arrows mark the positions of individual guttae, demonstrating consistent identification across corresponding DIC and autofluorescence images.

DM samples were obtained from FECD patients during DMEK surgery, and control specimens were derived from donor corneas deemed unsuitable for transplantation due to non-disease-related factors. In flat-mounted DM samples from FECD patients, guttae were prominently visible using differential interference contrast (DIC) microscopy (Fig. 1A) and striking structural differences were observed between central and peripheral guttae.

Peripheral guttae appeared as smooth, circular protrusions of relatively uniform size, distinctly elevated above the DM surface (DIC creates a spatial aspect making guttae appear to rise above the DM surface , Fig. 1A). While some peripheral guttae displayed a lobular contour or a flat border, most exhibited a rounded and sharply demarcated morphology. In contrast, central guttae seemed to be less elevated and were surrounded by fibrillar material that often extended over their surfaces, resulting in a blurry appearance. Large central guttae featured a well-defined concentric ring at their periphery (inset of Fig. 1A), a feature absent in peripheral guttae. These structural differences were discernible with phase contrast microscopy (suppl. Fig. S1), but DIC provided superior resolution of morphological details.

Guttae emitted green autofluorescence when excited at 480 nm (Fig. 1A), which precisely colocalized with the guttae visualized by DIC. In comparison, autofluorescence excited at 365 nm was considerably weaker. Interestingly, guttae with stronger green autofluorescence were associated with a more pronounced elevation above the DM surface in DIC imaging, whereas those with a fainter autofluorescence appeared less elevated. This correlation between autofluorescence intensity and topographical elevation suggests that autofluorescence intensity may reflect different developmental stages of guttae. Guttae with low autofluorescence and limited elevation often exhibited larger diameters, potentially representing early-stage guttae formed by large CEnCs.

While most guttae displayed a circular shape, elliptical and oval guttae were occasionally observed (Fig. 1A, confer suppl. Fig. S2). Similarly, although most peripheral guttae maintained sharply demarcated circular borders, some presented lobular or irregular contours.

To mimic the clinical aspect e.g. in slit lamp microscopy, a whole excised cornea was imaged from the epithelial side. Guttae could be detected both by DIC and autofluorescence imaging (Fig. 1B). However, DM flat-mounts provided significantly greater morphological detail. DIC imaging, in particular, proved optimal for thin samples, enabling clear visualization of subtle differences in guttae structure.

**Fig. 2:**
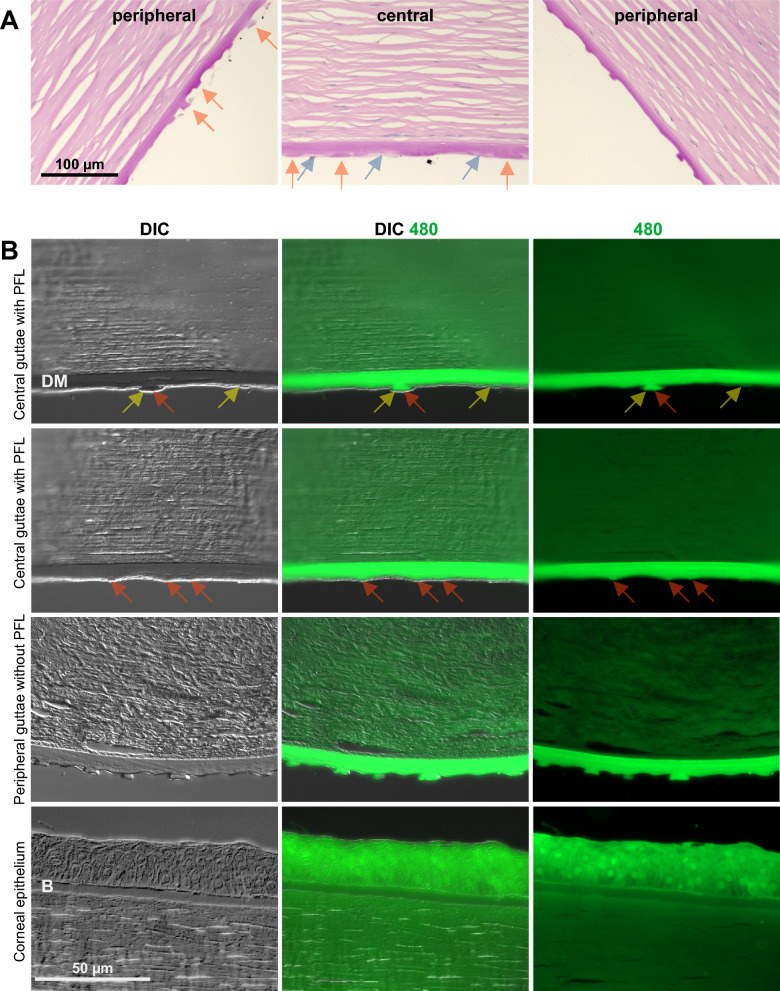
Peripheral and central guttae in cross-sections. (**A**) Periodic acid-Schiff (PAS) staining of a corneal cross-section, highlighting Descemet membrane (DM) with cross-sectioned guttae. The central DM is overlaid with a posterior fibrillar layer (PFL, blue arrows), which both surrounds and covers the guttae (red arrows). Refer to suppl. Fig. S3 for images of the whole cornea. (**B)** Green autofluorescence observed in unstained paraffin sections of a cornea. Autofluorescence is prominently localized within the DM and extends into the guttae (red arrows). While the PFL (yellow arrows) was detectable on the posterior layer overlaying a central gutta using DIC imaging, it did not exhibit green autofluorescence. The corneal epithelium is shown for comparison. It does not show a PFL and has much weaker autofluorescence. B indicates Bowman’s layer (anterior limiting membrane).

Corresponding structural differences between central and peripheral guttae were observed in paraffin sections of corneas from FECD patients. Periodic Acid-Schiff (PAS) staining revealed that peripheral guttae were distinctly elevated, whereas central guttae were flattened and covered by a fibrillar layer (Fig. 2A, suppl. Fig. S3). Central guttae often exhibited a mushroom-like structure, with a slender stalk at the base and a broadened cap covering the top.

Unstained paraffin sections revealed strong autofluorescence throughout the DM, including guttae (Fig. 2B). Interestingly, DIC imaging showed a thin superficial layer covering central guttae. This layer lacked autofluorescence and was absent in peripheral guttae. In contrast, the corneal epithelium was not covered by a superficial layer and exhibited weaker autofluorescence.

### Central guttae are covered by a fibrillar layer (PFL) containing COL1

**Fig. 3:**
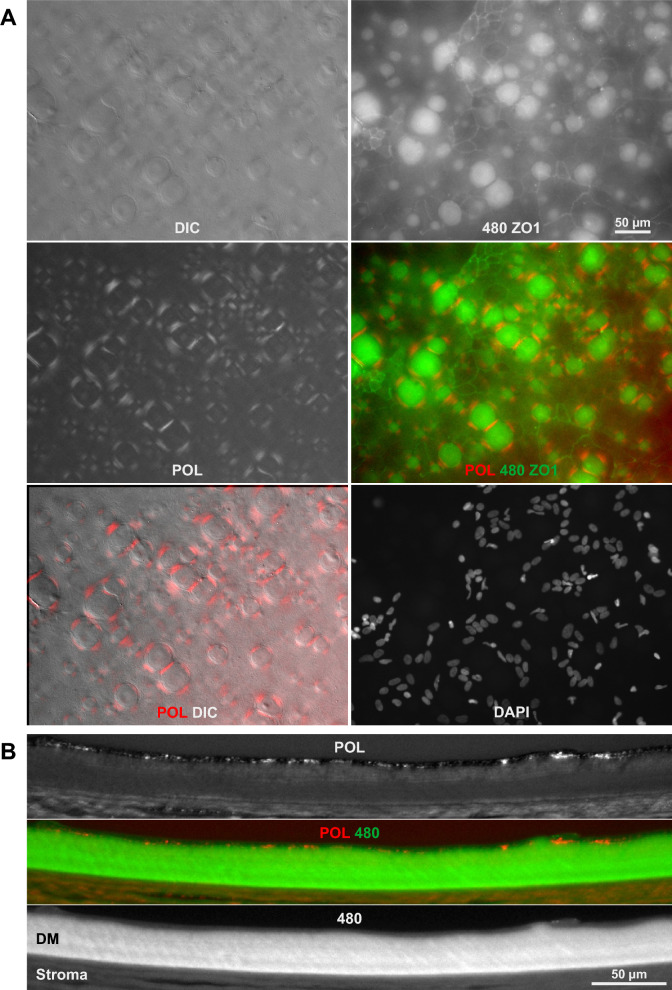
PFL with central guttae in polarized light. (**A**) Flat-mounted Descemet membrane (DM) from a central region exhibiting guttae. Polarized light microscopy (PLM) revealed faint polarization patterns surrounding the guttae, while the guttae themselves lacked a polarization signal. Under crossed polarization filters, light is suppressed totally. Collagen is rotating the polarization plane of the light so that it gets visible. The orientation of the faint parallel stripes (the polarization pattern of the collagen layer) depends on the orientation of the polarization filters in relation to the DM. Therefore, if the DM is rotated, the faint parallel stripes will also rotate indicating an unordered collagen mesh. Immunostaining for ZO1 highlighted the membranes of remaining corneal endothelial cells (CEnCs). (**B**) Polarization signals were also observed in the central region of unstained paraffin sections of cornea using PLM. Additional polarization was detected in the corneal stroma, indicating structural features of stromal collagen.

The fibrillar layer covering central guttae, as observed with DIC microscopy, suggested the presence of polarizing material that might be detectable using polarized light microscopy (PLM). Indeed, polarized light revealed a faint but distinct signal corresponding to the fibrillar layer detected in DIC (Fig. 3A). This signal displayed a characteristic pattern of two perpendicular pairs of short lines surrounding each gutta, a pattern typical for birefringent rings formed by polarizing material. The observed pattern rotated on a rotating stage, confirming its circular and birefringent nature. These polarization signals consistently surrounded the guttae as identified by autofluorescence or DIC. In unstained paraffin sections, the same polarized signal localized to the superficial layer of the DM (Fig. 3B).

**Fig. 4:**
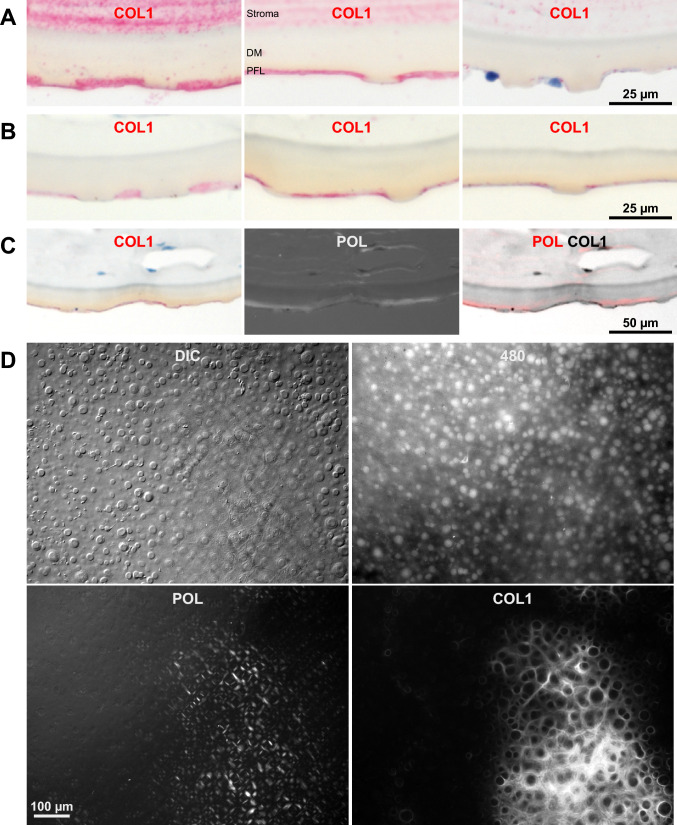
COL1 is found in the PFL of central guttae. (**A**) The left and middle images show COL1 staining (red, polyclonal antibody from Rockland) of paraffin sections of corneas with guttae localized to the posterior fibrillar layer (PFL) surrounding central guttae as well as to the corneal stroma. The right image shows peripheral guttae without PFL but some remnants of CEnCs (blue is nuclear counter staining). (**B**) Paraffin sections of corneas with guttae stained for COL1 (red, antibody from Abcam) revealed staining localized to the PFL surrounding central guttae. Low concentration of the monoclonal antibody resulted in a highly specific signal. (**C**) Image of a paraffin section as in B. The COL1 signal co-localized with polarization signals observed under polarized light (POL). Unfortunately, the middle part of the image contains a fold in the section. (**D**) Flat-mounted Descemet membrane (DM) stained for COL1 is depicted using differential interference contrast (DIC), autofluorescence, and polarized light (POL). Regions with COL1 staining exhibited corresponding polarization signals and a fibrous structure visible in the DIC image. See also suppl. Fig. S4.

As previous studies reported that the PFL contains collagen type 1 (COL1)^1,2,8^ we investigated COL1 staining in DM specimens. Consistent with earlier reports, COL1 staining of paraffin sections demonstrated a strong signal in the PFL, particularly within the spaces between central guttae (Fig. 4A, B). This COL1 staining colocalized with the birefringent signal observed under polarized light (Fig. 4C). Similar results were obtained in flat-mounted DM samples, where the COL1 staining corresponded to both the birefringent signal and the fibrillar layer visualized with DIC (Fig. 4D, suppl. Fig. S4).

**Fig. 5:**
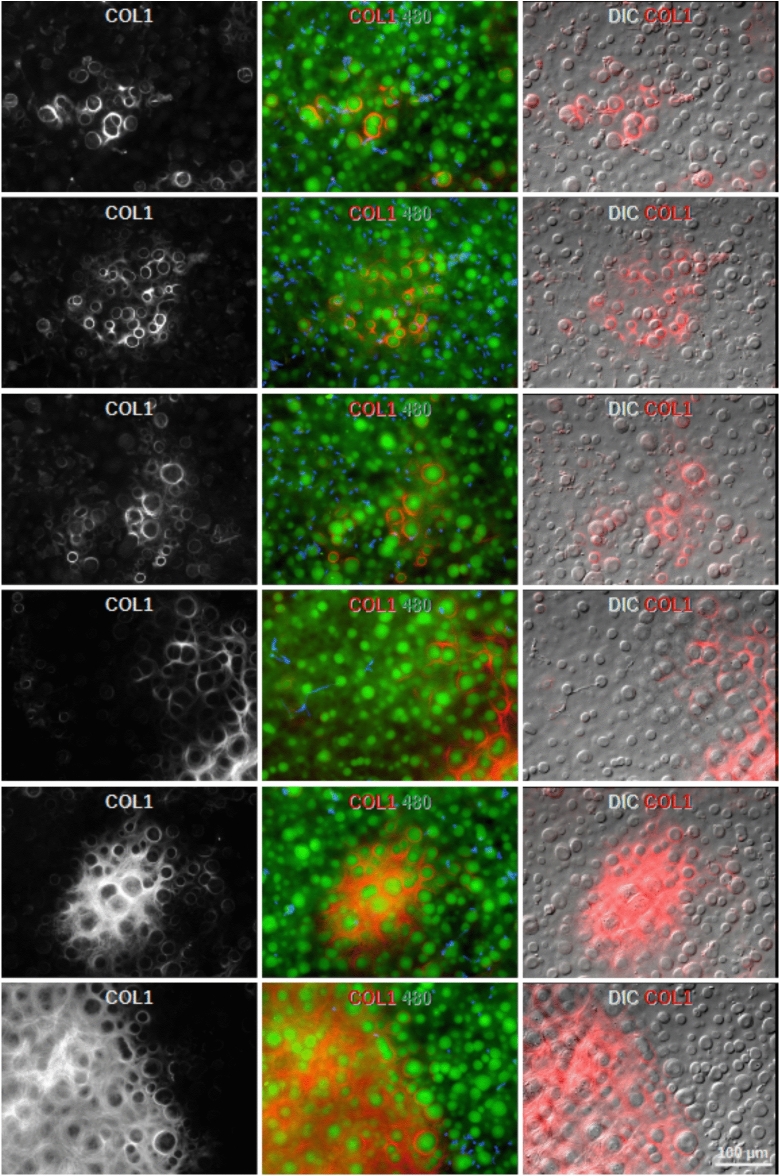
Stages of COL1 expression. Flat-mounted Descemet membrane (DM) stained for COL1 (red) demonstrates the progression of COL1 expression around guttae. Initially, COL1 staining is localized around individual guttae. In more advanced stages, COL1 deposits enlarge and eventually become confluent, forming a thin layer that surrounds and covers central guttae. Consequently, the posterior fibrillar layer (PFL) is never a uniform linear structure. See also suppl. Fig. S4 and suppl. movie 1.

The spatial distribution of COL1 across an entire DM is illustrated in suppl. Fig. S5. Areas of strong COL1 expression were characterized by central guttae surrounded by abundant collagen fibers, which frequently extended as a thin layer covering the surface of some guttae (Fig. 5, suppl. Fig. S4). At the periphery of these regions, guttae were encircled by distinct rings of COL1. In some cases, these rings exhibited asymmetrical staining, with the strongest signal localized near the fibrillar region (e.g. gutta near the center of the image in the third line of Fig. 5).

When two or more guttae were closely adjacent, they were often enclosed by a shared envelope of COL1, which formed a tight boundary around the group. A three-dimensional perspective of this arrangement was captured using confocal Z-stack imaging (suppl. movie 1). These images revealed that the COL1 envelope encircled the stalk-like base of central guttae and extended to form a thin layer over their cap-like structures.

### Stages of guttae formation

**Fig. 6:**
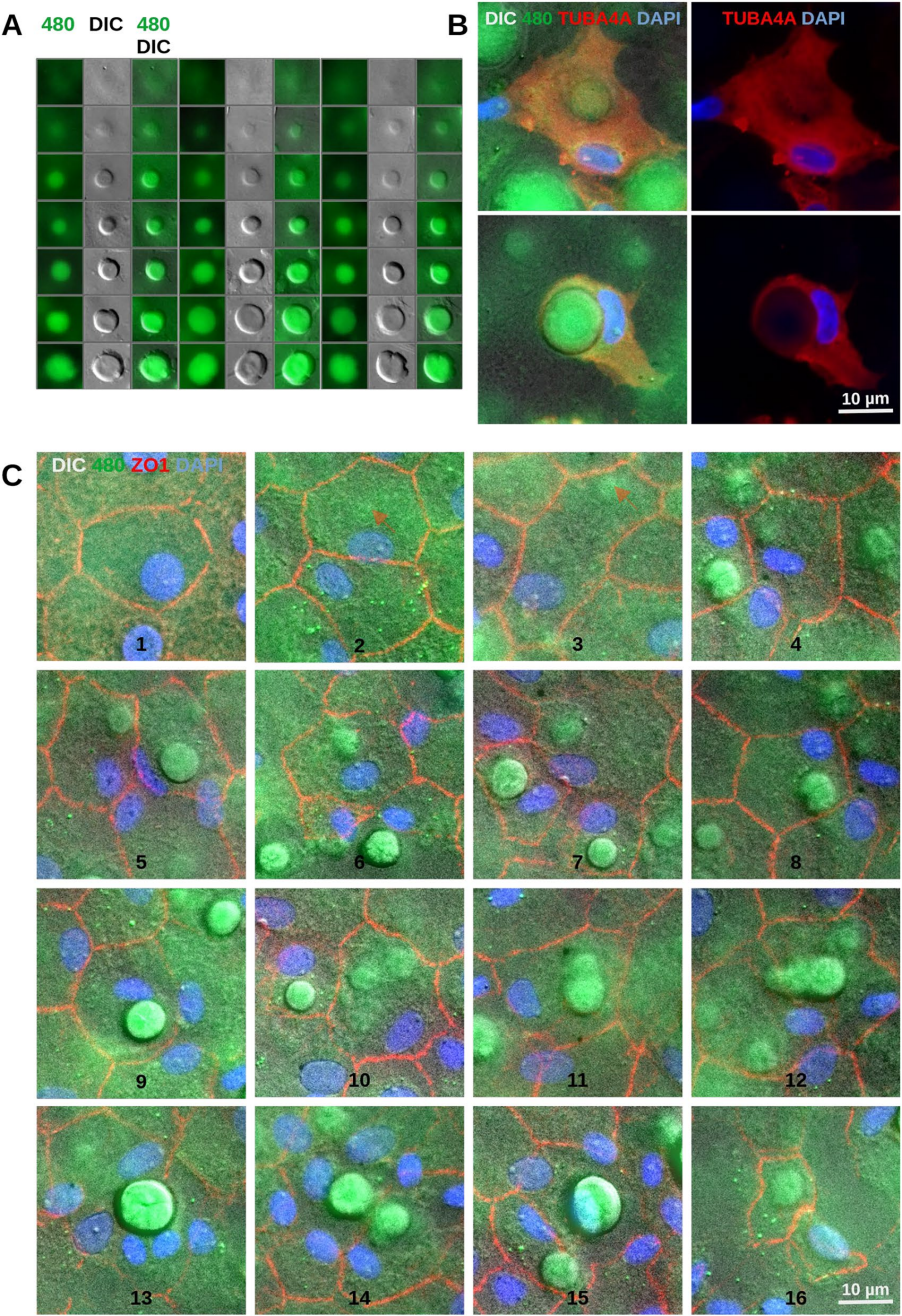
Different stages of guttae formation. (**A**) Guttae from a single flat-mounted Descemet membrane (DM) were arranged into three developmental series based on their elevation observed with differential interference contrast (DIC) microscopy and fluorescence intensity at 480 nm. Guttae were selected from areas without CEnCs. (**B**) Single CEnCs with guttae from flat-mounted DM stained for TUBA4A (red) to show the cell body. In the first line, a cell having a gutta with a small volume and little fluorescent material is shown. In the second line, a cell having a large gutta with the cytoplasm moved to one side is shown. Additional cells with more channel combinations are shown in suppl. Fig. S7. (**C**) Different stages of endothelial cell degeneration and guttae formation as observed from flat-mounted DM samples. Nuclei were stained with DAPI (blue) while cell borders were stained for ZO1 (red). Guttae are shown with autofluorescence at 480 (green) and DIC. 1. Healthy cells with circular nuclei. 2. Affected cells with elongated nuclei; the upper cell is initiating a gutta (arrow). 3. Cell with a small gutta (arrow). 4. Cell with a small gutta neighbored by cells with larger guttae. 5. Two cells with larger guttae. 6 - 8. Cells with larger guttae. 9. Cell with a large, prominent gutta. 10. Cell with two guttae alongside a cell with a large gutta. 11. Cell with two guttae. 12. Cells with several guttae and resolving cell borders. 13. Large gutta surrounded by nuclei of resolved cells. 14, 15. Guttae with resolving cells. 16. Gutta in a cell that lost the nucleus. Note that usually the nuclei were more stable than the ZO1 expression and were found as a remnant of the cells.

By combining both imaging modalities, even guttae with minimal elevation in DIC and low fluorescence intensity at 480 nm excitation were readily detected. Guttae from a single DM could be arranged into a developmental sequence based on their elevation and fluorescence intensity (Fig. 6A). This suggests that guttae form through continuous or stepwise secretion and deposition of material by CEnCs, resulting in progressive thickening of the DM at the sites of guttae formation. Importantly, these observations indicate that guttae within a single area of the DM often represent different stages of development (compare e.g. Fig. 1A or Fig. 5).

The extent of guttae formation and posterior fibrillar layer (PFL) deposition varied significantly across DMs from FECD patients. The most advanced progression of guttae formation typically localized to the central region of the DM flat-mount. Interestingly, even some control DMs (excluded from transplantation but used for research) exhibited a certain degree of guttae formation in the central cornea, though to a far lesser extent.

DMs from FECD patients were categorized in a developmental sequence based on the progression of guttae formation and PFL deposition (suppl. Fig. S6). Initially, in healthy regions where the endothelial cells had been mechanically removed, the DM surface appeared smooth and uniform, showing a grid-like pattern of faint elevations and depressions (suppl. Fig. S6B). The earliest observable stage of FECD involved the presence of small, isolated guttae covered by rather intact endothelial cells (suppl. Fig. S6C). The next stage showed larger and more elevated guttae still being overlaid by a continuous endothelial layer (suppl. Fig. S6D). Next, guttae were partially or fully covered by a PFL, with only remnants of endothelial cells remaining. In the final stages, a thicker PFL was found, usually with very few or no detectable CEnCs. Remnant CEnCs were found especially in peripheral regions.

The spatial distribution of guttae in relation to overlying cells was visualized in greater detail using immunostaining for ZO1 to highlight cell-cell borders or TUBA4A to reveal cytoplasmic microtubuli. This approach, combined with DIC and autofluorescence imaging, demonstrated that small guttae frequently localized to a more or less central region of individual CEnCs while some guttae were found at rather lateral positions (Fig. 6B and 6C, suppl. Fig. S6 and Fig. S7). Autofluorescent material appeared to form within the cell and secreted and deposited below the cell (towards the DM), often displacing the nucleus to an eccentric position. In later stages, cells were displaced to one side of a gutta, or the cell body appeared retracted from it, often leaving only the nucleus as a remnant.

### Hassall-Henle bodies

**Fig. 7:**
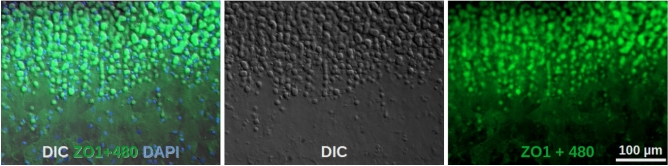
Hassall-Henle bodies. Flat-mounted DM was stained for DAPI and ZO1. Hassall-Henle bodies are located at the very border of the cornea near the trabecular meshwork. They look very similar to guttae having the same size and the same appearance both in DIC and in 480 nm autofluorescence. The lower end of the image is towards the corneal center. Though the DAPI staining showed the presence of nuclei the missing ZO1 staining indicated that CEnCs were not living anymore.

A special type of guttae, known as Hassall-Henle bodies, is found at the very border of the cornea next to the trabecular meshwork (Fig. 7). Due to this very peripheral position these bodies were only very rarely observed in our specimens. When present, they were separated from the peripheral guttae by a circular zone completely devoid of any guttae. In terms of size and appearance, Hassall-Henle bodies closely resembled peripheral guttae, including autofluorescence at 480 nm excitation. Of note, the image shown has a high density of Hassall-Henle bodies that were no longer covered by living cells, in contrast to the adjacent corneal area (not shown in the image). This suggests that the presence of Hassall-Henle bodies is not indicative of a healthy state of CEnC.

### Quantitative features of guttae

**Fig. 8:**
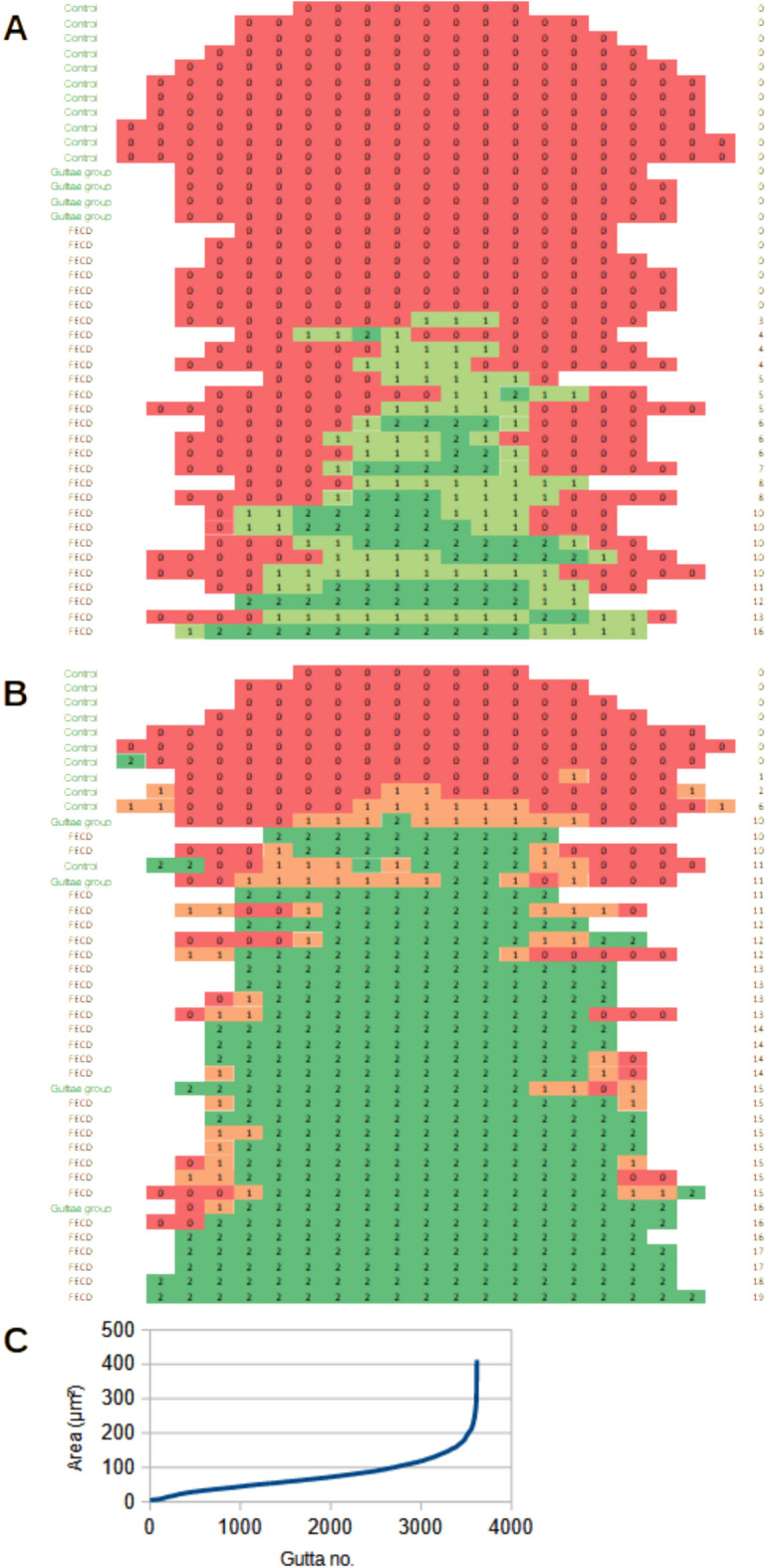
Quantification of PFL and guttae (**A**)  A series of adjacent images (DIC and autofluorescence) was captured from each DM flat-mount along one diameter. Each DM is shown in one line (peripher – central – peripher) and each box represents one image. For each image, the presence of the PFL was scored as follows: 0 for no PFL, 1 for partial PFL coverage, and 2 for complete PFL coverage. The resulting PFL scores (right column) were organized by the number of images showing at least part of the PFL. As expected, none of the control DM samples showed a PFL, while the majority of FECD DM samples displayed the PFL, usually in the central region of the DM. (**B**) A similar evaluation was performed on the same images for guttae count per image: 0 for no guttae, 1 for 1-10 guttae, and 2 for more than 10 guttae. Interestingly, while some control DM samples exhibited a considerable number of guttae, all FECD DM samples showed a significant number of guttae. Notably, in some FECD samples (lines 7 and 14), guttae number increased at the very periphery indicating Hassell-Henle bodies. (**C**) Histogram showing the size distribution of guttae. Thirty-two images from 8 DM specimens were evaluated. The mean number of guttae per image was 113. The maximum area of a gutta was 360 µm² (corresponding to a diameter of 21.4 µm), while the median area was 66.4 µm² (corresponding to a diameter of 9.2 µm).

In 43 DM flat-mounts, a series of adjacent images was captured along a diameter spanning from one end of the specimen to the other. This provided a detailed overview of the position and distribution of both peripheral and central guttae (examples are presented in suppl. Fig. S2). Some even included Hassall-Henle bodies at the specimen edge. These image series, obtained from multiple patients, were analyzed for the presence of guttae and the posterior fibrillar layer (PFL). While none of the control DM samples displayed any PFL, nearly all FECD patient samples (22 of 28) exhibited a PFL (Fig. 8A). The extent of PFL coverage varied widely, ranging from small, localized regions to cases where the entire DM area was affected.

All FECD-derived DMs, along with 5 of 11 control DMs, contained guttae (Fig. 8B). An additional four DMs from cornea donors were included in the analysis because slit lamp examination had identified guttae (referred to as the “guttae group”). Control corneas deemed unsuitable for transplantation – primarily due to insufficient CEnC density – frequently exhibited guttae, underscoring the relevance for this pre-operative investigation. In 17 of the FECD patient samples, guttae were observed across the entire DM surface.

Guttae density was determined in 105 images (DIC and autofluorescence) of DM flat-mounts, revealing a maximum density of 3500 guttae/mm^2^. To evaluate the guttae size, 32 representative images of 8 DMs were analyzed, yielding a mean count of 113 guttae per image. The largest individual guttae measured up to 360 µm^2^ in area, corresponding to a diameter of 21.4 µm. The median guttae area was 66.4 µm^2^ (25th quartile: 41.5 µm^2^, 75th quartile: 100 µm^2^), equating to a median diameter of 9.2 µm. These findings demonstrate a wide variation in guttae size, with only a few reaching large dimensions (Fig. 8C). Using the median guttae area and the maximal density observed, it was estimated that guttae could cover approximately 23.5% of the DM surface.

### Correlation with clinical patient state

Baseline patient characteristics are summarized in Table 1. To evaluate the relationship between histological findings and clinical features, two scoring systems were developed: a PFL score, representing the grade of posterior fibrillar layer (PFL) formation, and a guttae score, reflecting the extent of guttae distribution across each specimen (Fig. 8).

**Table 1:** Patient baseline characteristics.

**Characteristics**		N
Eyes	21 (100)	
Age [years]	71.0 [65.0 - 76.0]	21
Gender [female]	15 (71.4)	21
Type of DMEK [T-DMEK]	18 (85.7)	21
**Clinic**		
Visual acuity [ETDRS letters]	70.0 [ 66.0 - 78.0]	21
Glare [LogS]	1.5 [ 1.5 - 1.6]	16
Acuity factor [V-FUCHS]	0.9 [ 0.1 - 1.1]	14
Glare factor [V-FUCHS]	0.7 [ 0.4 - 1.0]	14
Visible edema [Krachmer grade 6]	9 (42.9)	21
**Scheimpflug imaging**		
Central corneal thickness [µm]	604.0 [575.0 - 622.0]	19
Anterior scatter [SU]	1932.3 [1593.6 - 2224.0]	18
Posterior scatter [SU]	1088.7 [954.1 - 1226.9]	18
Corneal edema [um]	92.0 [60.0 - 139.0]	17
Predicted edema resolution [µm]	84.1 [71.0 - 95.3]	18
**Laboratory**		
PFL score (sum of images with fibrosis)	6.0 [4.0 - 10.0]	21
Guttae score (sum of images with guttae)	15.0 [13.0 - 16.0]	21
Central guttae density (count per mm^2^)	2473 [1900 - 3387]	19

Data is presented as median and interquartile range or as count and percent of all observations (N). Fibrosis was graded as 0 = none, 1 = part of image, 2 = whole image and the value was added up in up to 20 images with a 1 or 2 grading. Guttae were graded as 0 = none, 1 = 1-10 guttae, 2 = >10 guttae per image and the value was added up in up to 20 images with a 1 or 2 grading.

The guttae score was calculated by summing areas with confluent guttae across the DM. However, no significant correlation was observed between the guttae score and clinical features. This likely reflects the early onset of guttae formation and their widespread distribution throughout the DM. While the presence of guttae is a sensitive marker for FECD detection, it lacks specificity as an indicator of disease severity, particularly in advanced stages.

**Fig. 9:**
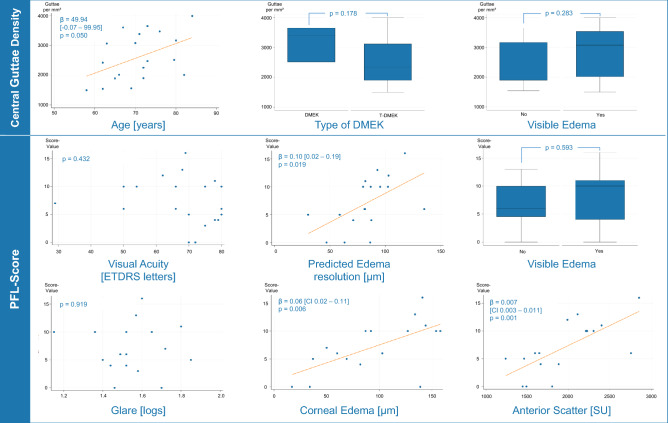
Correlation of Central Guttae Density and PFL score with clinical data. Central guttae density defined the number of guttae per mm^2^ in the central picture. It correlated positively with age. However, no correlation was found with other clinical characteristics such as type of DMEK (DMEK = DMEK only versus T-DMEK = DMEK with concomitant cataract surgery) or significant edema. The PFL score, as obtained in Fig. 8A, showed a significant correlation with anterior scatter, corneal edema, and predicted corneal edema resolution^4^. However, no correlation was observed between the PFL score and visual acuity, glare, or visible edema (Krachmer grade 6) in this small sample.

In contrast, the PFL score, which quantifies areas of PFL formation, demonstrated significant correlations with multiple clinical features. Since PFL formation progresses in later stages of FECD, the PFL score serves as a valuable indicator of disease severity. Notably, there was a robust correlation between the PFL score and anterior scatter measurements: for every 1000 units increase in scatter, the PFL score increased by 7 points (95% confidence interval [CI]: 3–11, p = 0.001; Fig. 9).

Additionally, the PFL score correlated with both corneal edema resolution and predicted edema resolution^4^ following DMEK:

- For every 10 µm increase in predicted corneal edema resolution (predicted edema resolution), the PFL score increased by 1 point (95% CI: 0.2–1.9, p = 0.019; Fig. 9).
- For every 10 µm increase in actual corneal edema resolution (corneal edema), the PFL score rose by 0.6 points (95% CI: 0.2–1.1, p = 0.006; Fig. 9).

Central guttae density was high in all FECD membranes. Similar to the guttae score, it did not correlate with clinical parameters but showed a positive correlation with age. Specifically. central guttae density increased by 50 guttae per mm^2^ for each year of age (95% CI: -0.1–100.0, p = 0.050, Fig. 9).

These findings highlight the potential of the PFL score as a robust indicator for disease severity staging in advanced FECD. Unlike the guttae score, the PFL score provides a specific measure that correlates strongly with clinical parameters associated with disease progression. Central guttae density appears to be increased with age, even in advanced FECD, while not correlating with clinical features – likely because age-related progression is modest compared to the total burden of preexisting guttae.

## Discussion

This study highlights the effectiveness of DIC microscopy for detailed visualization of DM, including guttae and the PFL. Guttae exhibited strong autofluorescence under 480 nm excitation, while the PFL was distinctly visible in PLM. Notably, the PFL correlated with clinical parameters such as anterior scatter and corneal edema, thereby bridging clinical and histological assessments.

The presence of guttae in FECD was first described over a century ago^9^ and has since been extensively investigated, primarily using slit lamp examination and electron microscopy. These studies provided valuable insights into the ultrastructure of guttae, with occasional references to a fibrillar layer^1–3,8,10,11^. Our findings build on this body of work by demonstrating that the PFL can be observed in its full areal extent using DIC in flat-mounted DM specimens. This approach revealed distinct stages of PFL development within individual specimens and across patients, providing a more comprehensive understanding of the pathological progression of FECD.

The association of PFL formation and CEnCs loss suggests that the PFL represents a terminal stage of CEnC decomposition. Previous electron microscopy studies suggested that the PFL is primarily composed of fibrillar collagen^1^, and our study confirms this hypothesis by demonstrating the presence of COL1 in the PFL, especially between guttae. Interestingly, the collagen layer forms a dense cover over central guttae and transitions into discrete, circular structures surrounding individual peripheral guttae. Overlays of immunofluorescence images with DIC and autofluorescence images proved invaluable for accurately localizing guttae in relation to collagen deposition. Corresponding structures were identified in a previous study, where collagen was detected by second harmonic generation (SHG) microscopy and guttae were visualized with fluorescence excited at 740 nm^12^. However, this technique did not allow for differentiation between collagen types.

PLM further confirmed the PFL’s collagenous composition, with observed birefringence patterns consistent with collagen fibrils^13^. The polarized light signal encircled guttae, while their centers remained dark, a pattern confirmed through image overlays with autofluorescence and DIC. These findings suggest that PLM is an additional robust technique for detecting and studying the PFL, particularly in flat-mounted specimens. Further studies may explore whether polarized light can be adapted for clinical diagnostics of diseases involving PFL formation.

The combination of DIC and 480 nm-excited autofluorescence provided unique insights in different stages of guttae formation. The strong autofluorescence observed in both DM and guttae aligns with previous reports^12,14,15^ and may be attributed to glycation of extracellular proteins, such as collagen, deposited by stressed CEnCs. Our results indicate that guttae formation is a dynamic process, likely involving the prolonged secretion and accumulation of autofluorescent material by stressed or dystrophic CEnCs as described elsewhere^16,17^. Early pathological changes in CEnCs include increased variability in cell size and the transition from circular to elongated nuclei. Subsequently, guttae with low fluorescence and minimal elevation form within individual cells. As guttae enlarge, nuclei and cell bodies are displaced laterally. Eventually, the cells resolve, often leaving remnant nuclei (Muir *et al*, 2021). It may be speculated that these remnant nuclei are a result of CEnCs dying by ferroptosis rather than apoptosis, as apoptosis typically involves nuclear degradation. Notably, small diameter guttae are formed by small cells rather than indicating an early developmental stage. In late stages, guttae may become capped by a collagen layer, particularly in advanced stages and at the central region of the DM, which may explain the characteristic circular distribution of COL1 observed on their surface (Fig. 5).

Attempts to culture CEnCs from DM in later disease stages with a PFL frequently fail^11^, suggesting that cells are more viable before PFL formation. Similarly, our results indicate a loss of CEnCs in areas with a PFL. Regions with high guttae density were also rarely covered by cells. These observations underscore the critical need for early nutritional or therapeutic intervention to preserve endothelial function.

In vivo imaging by confocal microscopy showed guttae diameter of 10 - 40 µm^18^ or 18 µm^14^. Our study with fixed and flat-mounted DM samples determined a median guttae diameter of 9.2 µm, which is somewhat smaller than previously reported values from fixed DM samples where guttae sizes showed a mean of 27 µm (range 4 - 80 µm)^19^. This discrepancy may reflect differences in patient populations or imaging methodologies, as our approach enables the detection of very small guttae. Fixation-induced shrinkage of the DM does not appear to be a major contributing factor as the values obtained from fixed DM are in the same range as those found by in vivo confocal microscopy, and guttae sizes showed a high variability. In fact, in a study using cat skin it was found that formalin fixation had almost no influence on sample diameter^20^. Neverthelesss, the ability to quantify guttae from flat-mounted DM in terms of density, area, and size distribution using DIC and autofluorescence offers new opportunities for standardized ex vivo analyses.

The density of healthy CEnCs has been reported as 3100 cells / mm^2^ in a Japanese population with a mean age of 19.6 years^21^. Our observed maximal guttae density of 3500 guttae / mm^2^, falls within the upper range of CEnC density and may indicate that, at least in some cases or areas, each CEnC is able to initiate a single gutta. Interestingly, it was found that DM shows a regular pattern of elevations and depressions at sites without guttae and cells, correlating with cell size (suppl. Fig. S6B). This may indicate that each CEnC contributes to the thickening of the DM over time and that enhanced localized secretion of material is a precursor of guttae formation. The arrangement of guttae in rows or even grids may further reflect their origin from individual cells.

 Traditionally, FECD staging has relied on parameters such as CEnC density, guttae density, and size, as well as the presence of a PFL. Early studies using specular microscopy^22^ described stages of guttae development that align closely with our observations. However, these structures, difficult to visualize clearly with specular microscopy but better resolved with transmission interference microscopy^23^, are readily identifiable with DIC, making it a superior technique for detailed analysis of DM pathology. While specular microscopy remains a valuable in vivo method, it requires a largely intact endothelial cell layer to achieve proper focus. In its absence, guttae or endothelial cells cannot be reliably imaged. Furthermore, specular microscopy typically provides only a random central view of the endothelium, which may not be representative of the entire cornea. Targeted selection of specific endothelial regions is not feasible with this method.

The clinical relevance of these findings is underscored by the strong correlation between PFL formation and disease severity. The PFL score, which quantifies the extent of fibrillar layer formation, demonstrated significant associations with anterior scatter and corneal edema (Fig. 9) — both established markers of advanced FECD severity^24,25^. In contrast, while the guttae score is highly sensitive for early disease detection, it lacks specificity for severity staging due to the widespread presence of guttae across all disease stages. Previous literature described different guttae morphologies linked to specific genetic causes of FECD, emphasizing their progressive enlargement and coalescence over time, further demonstrating that guttae diameter plays a critical role in endothelial cell function accelerating disease progression^15,16,19,26^. These findings support our conclusion that while the presence of guttae is a hallmark of FECD, their size and density vary with disease progression. In contrast, the PFL score may serve as a more reliable marker for staging advanced FECD and predicting clinical outcomes, including corneal edema reduction post-DMEK.

To improve the accuracy of the guttae score, it would be necessary to include images with low guttae counts at each end of the image series to ensure a representative assessment. Consequently, DMs with only high-density guttae images and limited coverage due to small excision diameters may yield artificially low guttae scores. Excluding these DMs did not significantly alter the guttae score, likely due to the limited number of usable specimens. Particularly in late-stage patients, excising larger diameters to capture peripheral regions would be ideal but is incompatible with optimal patient care. We also evaluated whether counting guttae in every image would improve the guttae score. However, neither total guttae counts nor maximal or median values produced a meaningful or reliable guttae score. Further research into a guttae score needs to find a way to standardize the number of counted images per specimen but with respecting every specimens individual characteristics.

In conclusion, this study demonstrates that DIC, PLM, and autofluorescence microscopy are powerful tools for studying the pathological changes in DM associated with FECD. These methods enable detailed visualization and quantification of guttae and the PFL, offering critical insights into their formation and progression. Furthermore, the correlation between PFL formation and clinical parameters highlights its potential as a diagnostic marker for assessing disease severity. Future research should focus on standardizing ex vivo grading methods for the PFL and guttae, which could enhance disease severity assessment and guide therapeutic strategies in FECD.

## Materials and methods

### Ethical statement

This prospective study was approved by the institutional ethics committee at the Faculty of Medicine Freiburg and conformed to the tenets of the Declaration of Helsinki. Donor screening and eligibility criteria followed the Freiburg Lions Eye Bank and European Eye Bank Association standards. All donors or their relatives gave written informed consent. All participants undergoing Descemet membrane endothelial keratoplasty (DMEK) were screened for eligibility for our prospective cohort study and asked to give written informed consent before inclusion.

### Human specimens

This study included participants with Fuchs’ endothelial corneal dystrophy (FECD) undergoing DMEK, with or without phacoemulsification and posterior chamber intraocular lens implantation, as described previously^27^. Only participants with a stable follow-up of at least three months after DMEK and high-quality Scheimpflug imaging before and after surgery were included. Exclusion criteria encompassed corneal comorbidities unrelated to FECD, as well as previous ocular surgeries other than uncomplicated cataract surgery or YAG-laser capsulotomy.

FECD severity was graded using slit lamp biomicroscopy based on the area and confluence of guttae and the presence of edema (visible edema), applying a modified Krachmer grading system^28^. Best-corrected visual acuity was assessed using Early Treatment of Diabetic Retinopathy Study (ETDRS) charts after standardized subjective refraction^29^. Participants completed the validated German version of the patient-reported visual disability V-FUCHS instrument, a patient-reported measure of visual disability, where lower scores indicate less disability^30^.

Scheimpflug imaging (Pentacam HR, Oculus, Wetzlar, Germany) was performed according to the manufacturer’s instructions. Corneal edema resolution (corneal edema) was calculated as the absolute difference between central corneal thickness before and after DMEK. Predicted corneal edema resolution (predicted edema resolution) was estimated preoperatively based on Scheimpflug imaging parameters, as described previously^4^.

Two types of Descemet membrane (DM) specimens were analyzed:

1. **DM from FECD patients**: Specimens were collected from 28 participants during endothelial keratoplasty. Surgeons excised the central 8 mm of DM using a Price hook. After extraction, specimens were transferred to balanced salt solution to allow unfolding and then fixed in formalin.
2. **DM from healthy donors**: Fifteen corneas with intact, healthy DM were obtained from the local eye bank. These donor corneas were deemed unsuitable for transplantation. Among these, four specimens exhibited guttae, which were identified during slit lamp examination at the eye bank (“guttae group” in Fig. 8). Experienced corneal surgeons stripped the central 8 mm of DM following previously established protocols^31^.

### Immunofluorescent staining of DM for flat-mount preparation

After isolating the Descemet membrane (DM), samples were stored in Tris-buffered saline (TBS) at 4 °C until further processing. Fixation was performed using 4% buffered formalin for 30 min at room temperature, followed by storage in TBS at 4 °C until immunofluorescence analysis.

Prior to antibody staining, DM specimens were blocked in a blocking solution containing 5% goat serum and 0.1% Triton X-100 in TBS. Blocking was carried out in 24-well plates at room temperature for 1 h. Specimens were then incubated with primary antibodies diluted in an antibody solution (1% goat serum and 0.1% Triton X-100 in TBS) for 1 h. The following primary antibodies were used:

- ZO1 (TJP1, rabbit, polyclonal, 1:200, #40-2200, ThermoFisher Scientific, Dreieich, Germany)
- ZO1 (TJP1, mouse, clone ZO1-1A12, 1:500, #33-9100, ThermoFisher Scientific, Dreieich, Germany)
- COL1 (COL1A1, rabbit, clone EPR7785, 1:500, #ab138492, Abcam, Cambridge, UK).

After primary antibody incubation, the DM was washed three times with TBS, 5 min per wash. Specimens were subsequently incubated for 1 h with secondary antibodies diluted in TBS, including:

- Anti-Mouse-AF488 (#715-545-151, Jackson Immunoresearch Europe, Ely, UK)
- Anti-Rabbit-AF568 (#A10042, Life Technologies, Karlsruhe, Germany)
- Anti-Mouse-AF647 (#115-606-146, Jackson Immunoresearch Europe, Ely, UK).

DAPI was included in the secondary antibody solution for nuclear staining. Following secondary antibody incubation, specimens were washed three times with TBS for 5 min each. The stained DM samples were then mounted using a fluorescence mounting medium.

In the first experiments, ZO1 was stained in the FITC channel (excited at 480 nm). After finding that guttae show a nice autofluorescence in this channel, it was omitted for staining.

### Immunohistochemistry

After extraction, DM were fixed immediately in 4% buffered formalin. Fixed samples were processed using established histopathological protocols at the Ophthalmopathological Laboratory of the Eye Center, University Medical Center, Freiburg.

Paraffin sections were stained using an automatic stainer (BenchMark GX, Roche Diagnostics, Mannheim, Germany). Antigen retrieval was performed by demasking with citrate buffer or Tris/HCl buffer (COL1 antibody from Rockland) for 30 min. The following primary antibodies were applied:

- COL1 (COL1A1, rabbit, clone EPR7785, 1:500, #ab138492, Abcam, Cambridge, UK).
- COL1 (COL1 + COL3, rabbit, polyclonal, 1:400, #600-401-103, Rockland, Limerick, USA).

Staining procedures adhered to the standard protocols of the BenchMark GX stainer, ensuring consistent immunohistochemical labeling of corneal tissue sections.

### Microscopy

Specimens were analyzed using various microscopy techniques to evaluate their structural and fluorescence characteristics:

- **Fluorescence microscopy**: Fluorescence was visualized using a fluorescence microscope (Zeiss Observer Z.1 or Zeiss AxioImager A.1, Zeiss, Oberkochen, Germany) with the following excitation/emission filter sets:
  o Excitation at 365 nm and emission at 445 nm.
  o Excitation at 480 nm and emission at 525 nm.
  o Excitation at 550 nm and emission at 605 nm.
  o Excitation at 640 nm and emission at 690 nm.
- **Differential interference contrast (DIC)**: Both microscopes were equipped with Nomarski optics to enable DIC imaging for enhanced structural contrast.
- **Polarized light microscopy (PLM)**: PLM was performed with the AxioImager using two polarization filters to visualize birefringent materials, such as collagen in the PFL. One of the polarization filters, the polarizer, was placed between the white light source and the condenser while the other, the analyzer, was situated between the objective and the eyepiece or camera resulting in a setting with linearly polarized light. Polarization was observed using a 90 ° crossed filter orientation.
- **Confocal microscopy**: A Leica TCS SP8 confocal microscope (Leica, Wetzlar, Germany) was used for high-resolution imaging of specific structures and fluorescent markers.

For precise image overlay, adjustments were made to align DIC images with fluorescence or polarized light images. DIC images required a shift of 4 pixels to the left and 4 pixels downward in most cases due to the orientation of the camera and the optical displacement caused by DIC prisms. To determine the necessary adjustments, identifiable small dots visible in both DIC and fluorescence images were used as alignment references.

### Quantification of guttae

A series of adjacent images along a complete diameter of the DM flat-mount was captured from all DM samples for analysis (compare suppl. fig. S2). Images included the following channels: DIC, autofluorescence, DAPI, and others.

- **Determination of PFL areas**: The presence of a Pre-Fibrillar Layer (PFL) was assessed using DIC images.
- **Guttae detection**: Guttae were quantified using autofluorescence images processed in ImageJ (https://imagej.net/). The following steps were performed:
- A bandpass filter was applied to exclude structures larger than 50 pixels or smaller than 10 pixels.
- The “Find Maxima” function was used to identify guttae, adjusting the prominence threshold between 3 and 11 to optimize detection and minimize background counts.
- **Guttae area measurement**: The area of guttae was calculated from autofluorescence images as follows:
- Background subtraction using a rolling ball radius of 50 pixels.
- Local thresholding using the Bernsen method with a radius of 15 pixels.
- Morphological adjustments with a dilation and erosion step, followed by hole filling.
- Particle analysis was performed, selecting particles larger than 50 pixels with a circularity between 0.6 and 1.
- **PFL and guttae scores**: Areas of PFL formation and areas with guttae were summed to generate a PFL score and guttae score, respectively. Partially filled areas (containing PFL or guttae only in part) were assigned a score of 1 while fully filled areas were assigned a score of 2. This scoring system enabled standardized quantification of PFL and guttae extent across specimens (Fig. 8).
- **Central guttae density**: The number of guttae per mm^2^ was calculated by manually counting all guttae in one image (image area = 0.15 mm^2^).

### Statistics

Descriptive statistics were used to summarize the data. Continuous variables were expressed as median and interquartile range (IQR), and categorical variables as counts and percentages. Differences in characteristics between different subgroups were analyzed using the Mann-Whitney U test for continuous variables and Pearson’s chi-square test for categorical variables as well as by multiple regression analysis. All statistical analyses were performed using Stata version 17.0 (StataCorp, College Station, TX, USA). A p value < 0.05 was considered statistically significant.

## Supplementary Information

Supplementary Information 1.

Supplementary Information 2.

Supplementary Information 3.

Supplementary Information 4.

Supplementary Information 5.

Supplementary Information 6.

Supplementary Video 1.

Supplementary Information 7.

## Data Availability

Data is provided within the manuscript or supplementary information files. Data used and analysed during the current study to optimize the procedures are available from the corresponding author on reasonable request.
