# Supplementary figures and images for: Imaging improvements reveal guttae development and posterior fibrillar layer formation in fuchs endothelial corneal dystrophy
Source: Sci Rep. 2026 Mar 26;16:10501. doi: 10.1038/s41598-026-44926-2 (PMC13031840; doi:10.1038/s41598-026-44926-2)

# Suppl. Fig. S1

Peripheral guttae

Central guttae

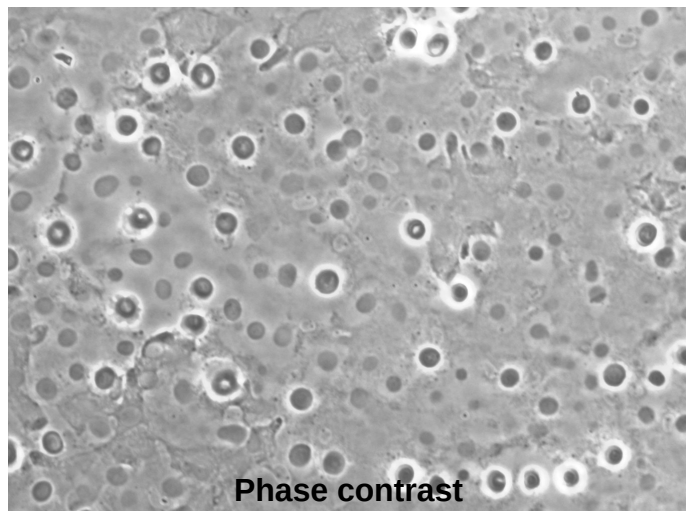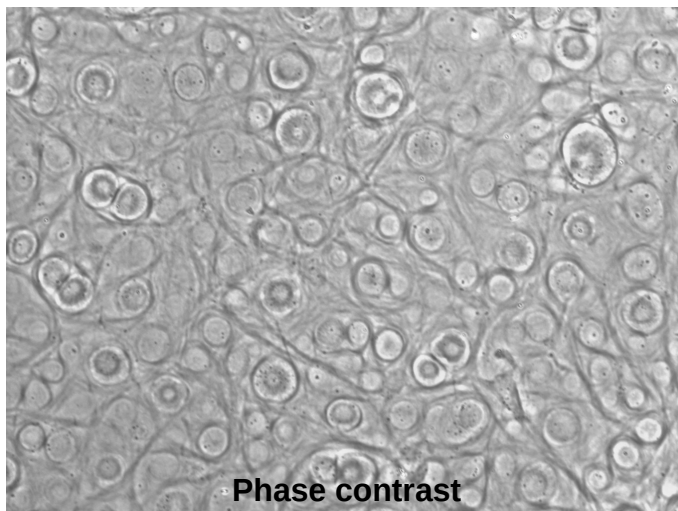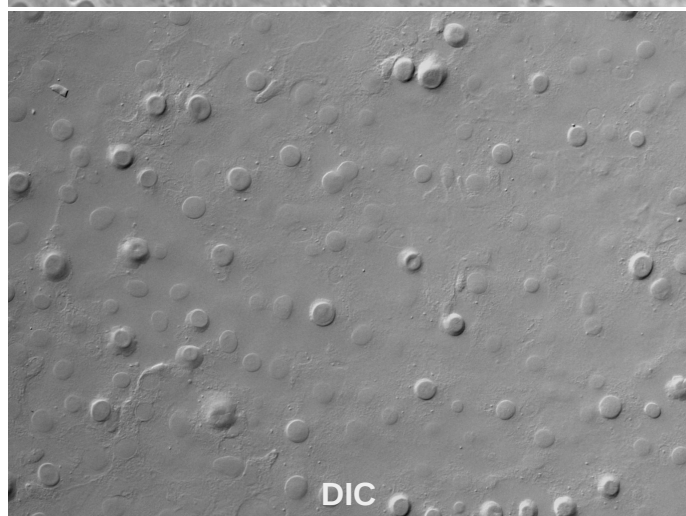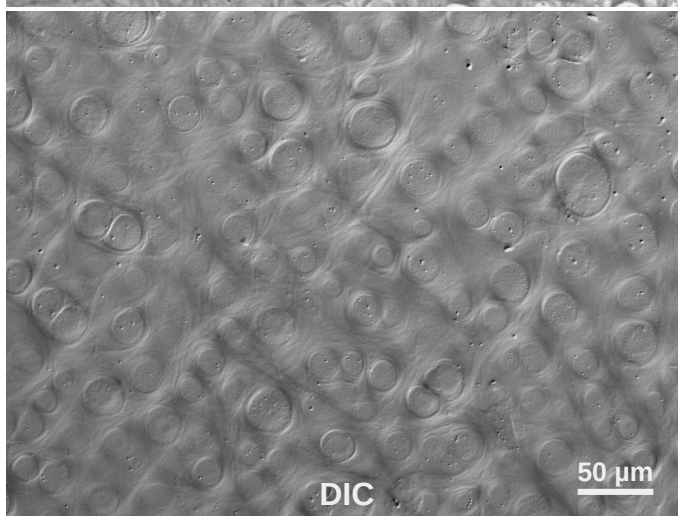

Suppl. Fig. S3

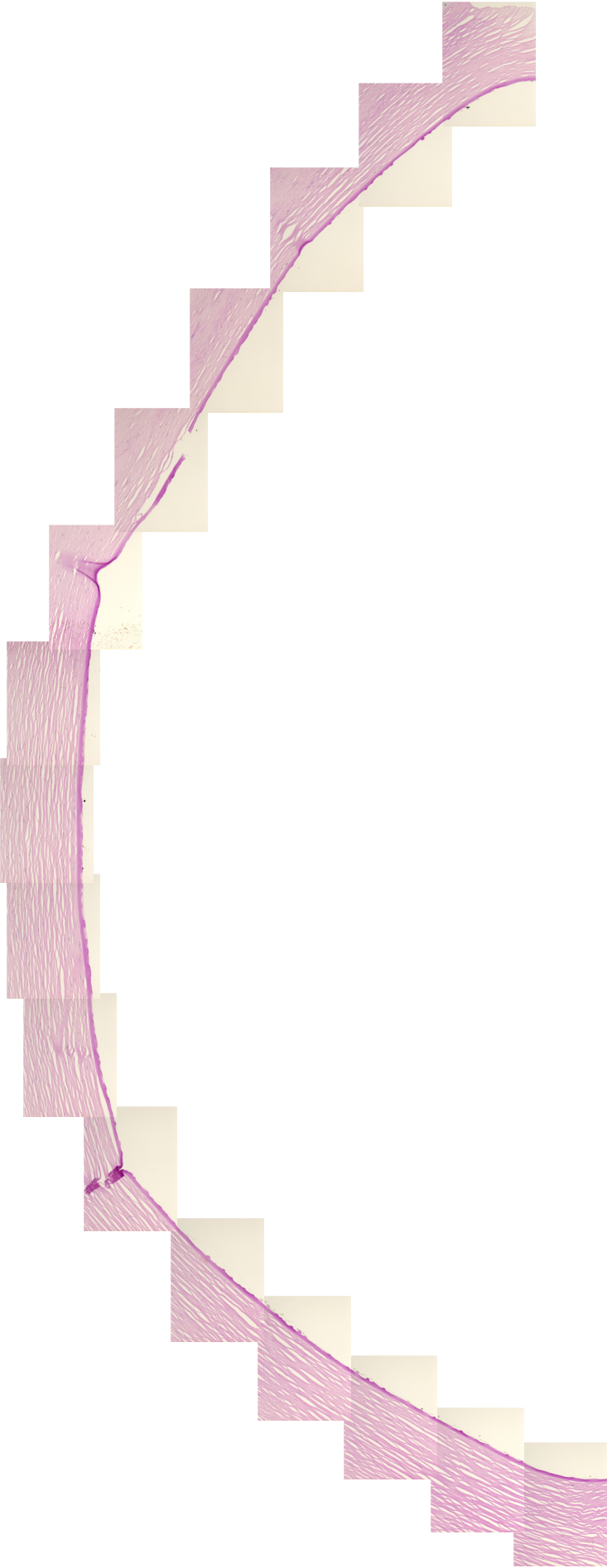

Supplement: Supplementary file 1 — Supplementary Information 1. [file 41598_2026_44926_MOESM1_ESM.pdf]

Suppl. Fig. S2

**A**

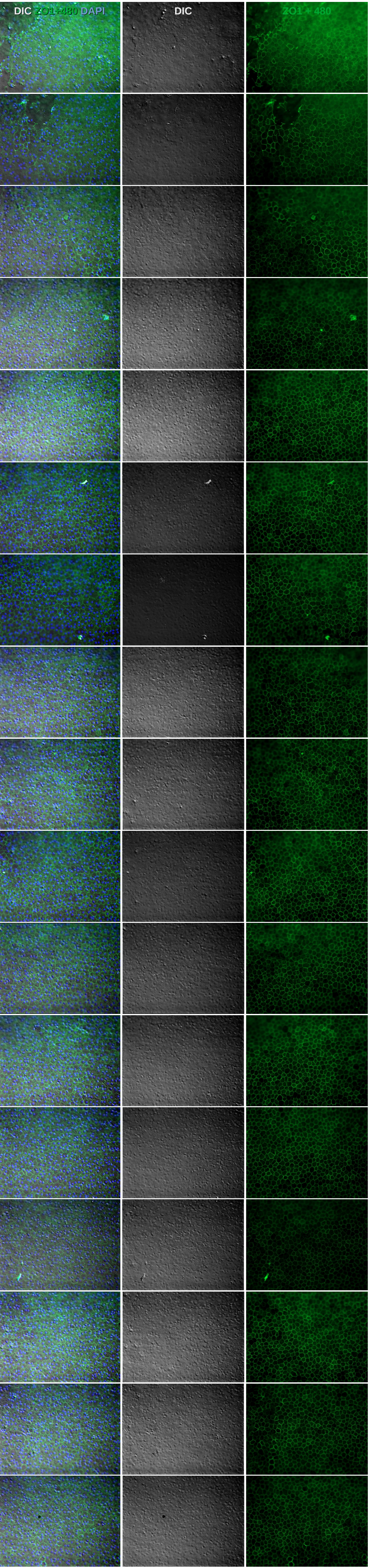

**B**

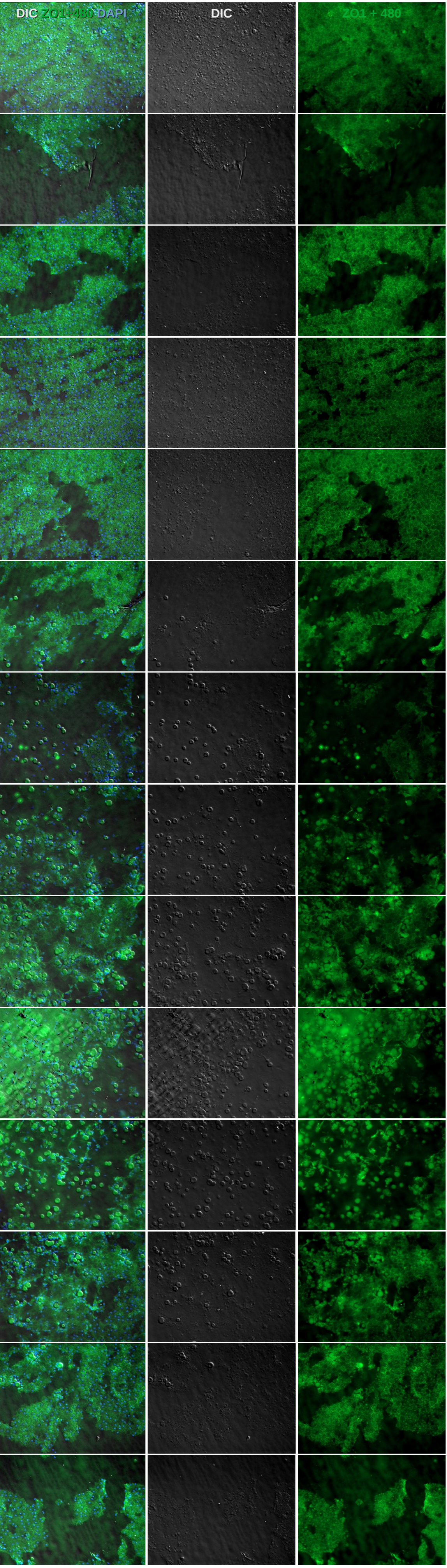

**C**

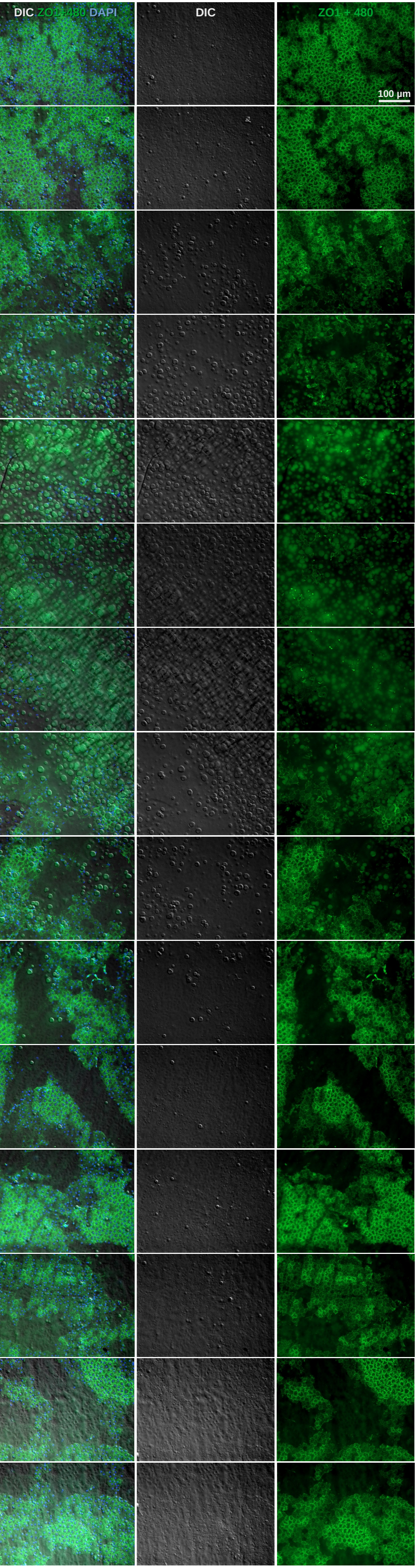

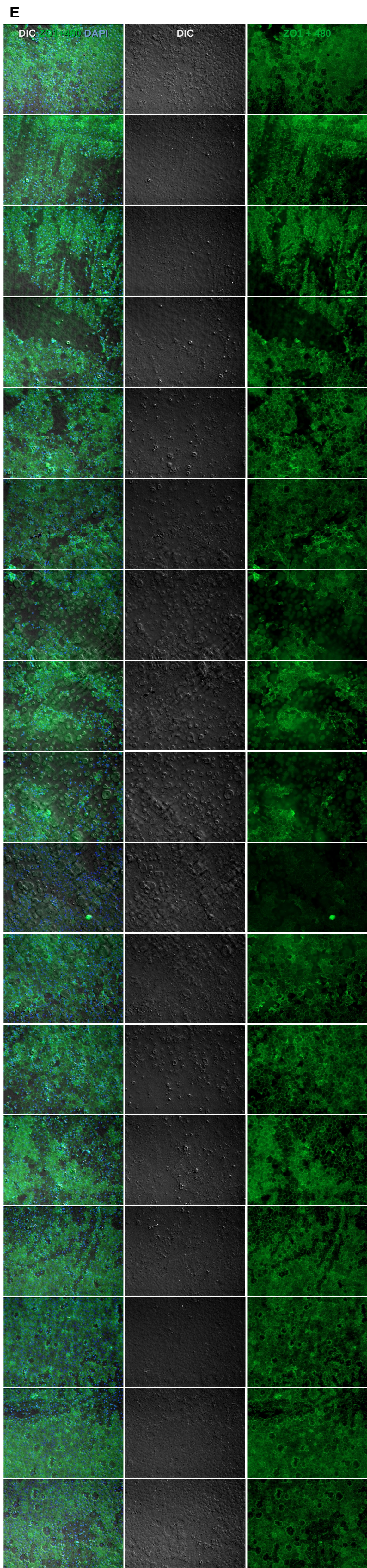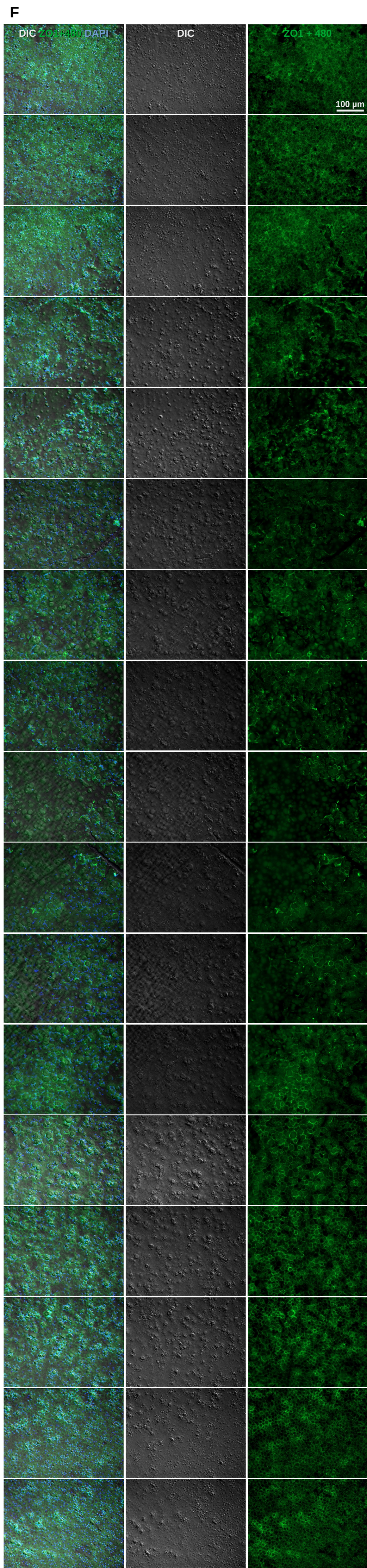

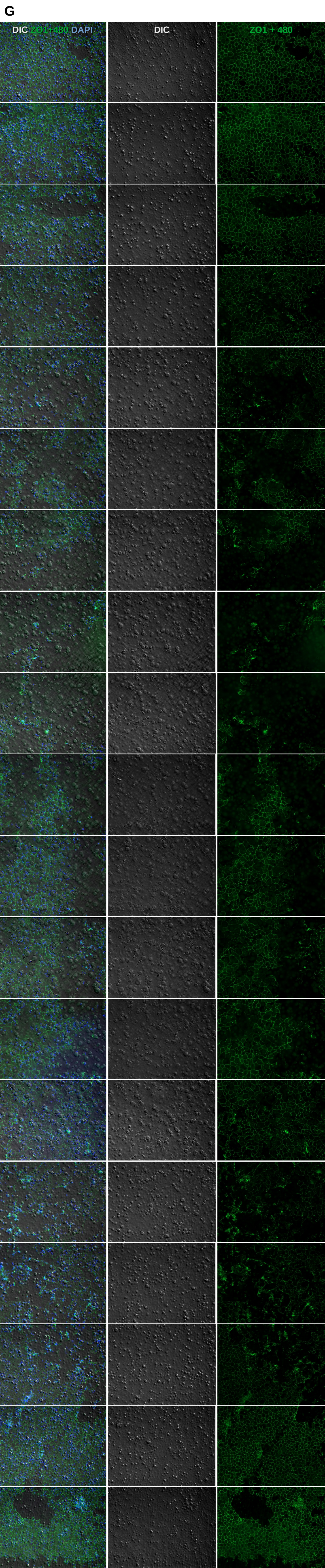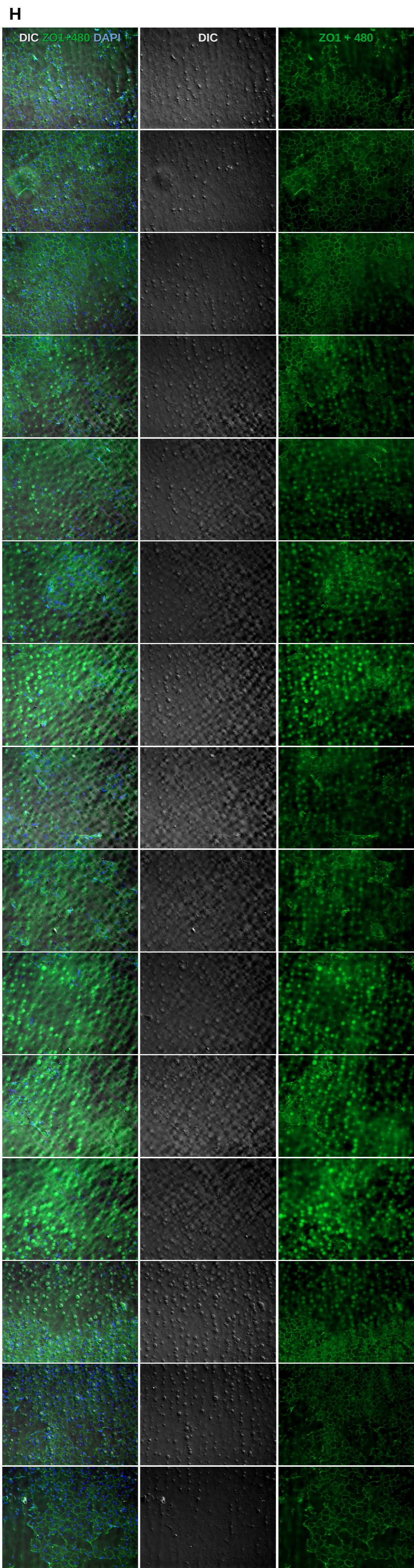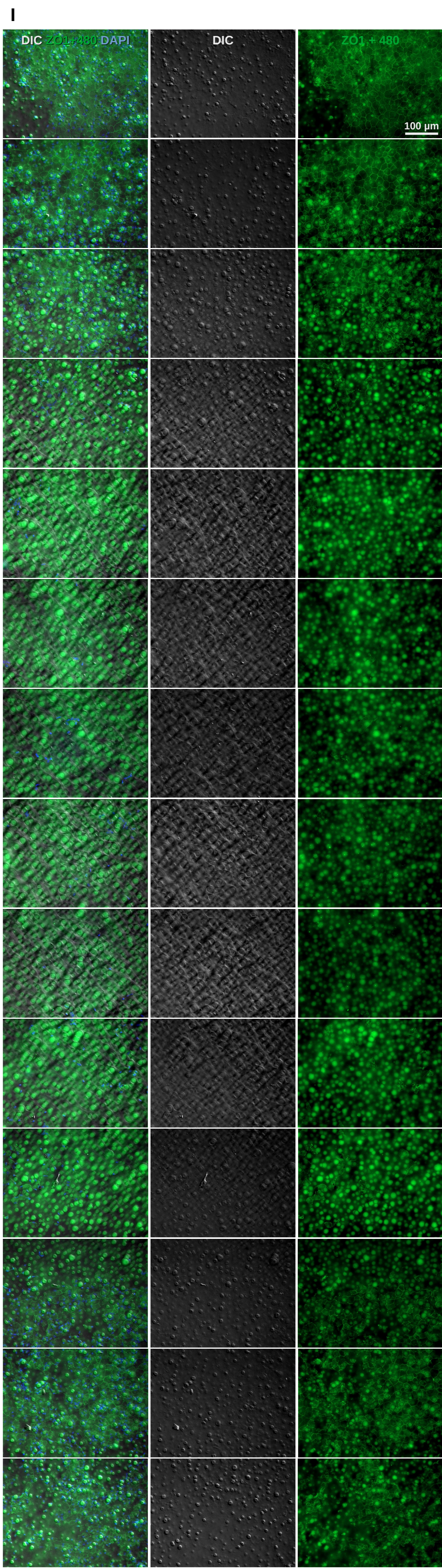

Supplement: Supplementary file 2 — Supplementary Information 2. [file 41598_2026_44926_MOESM2_ESM.pdf]

Suppl. Fig. S4

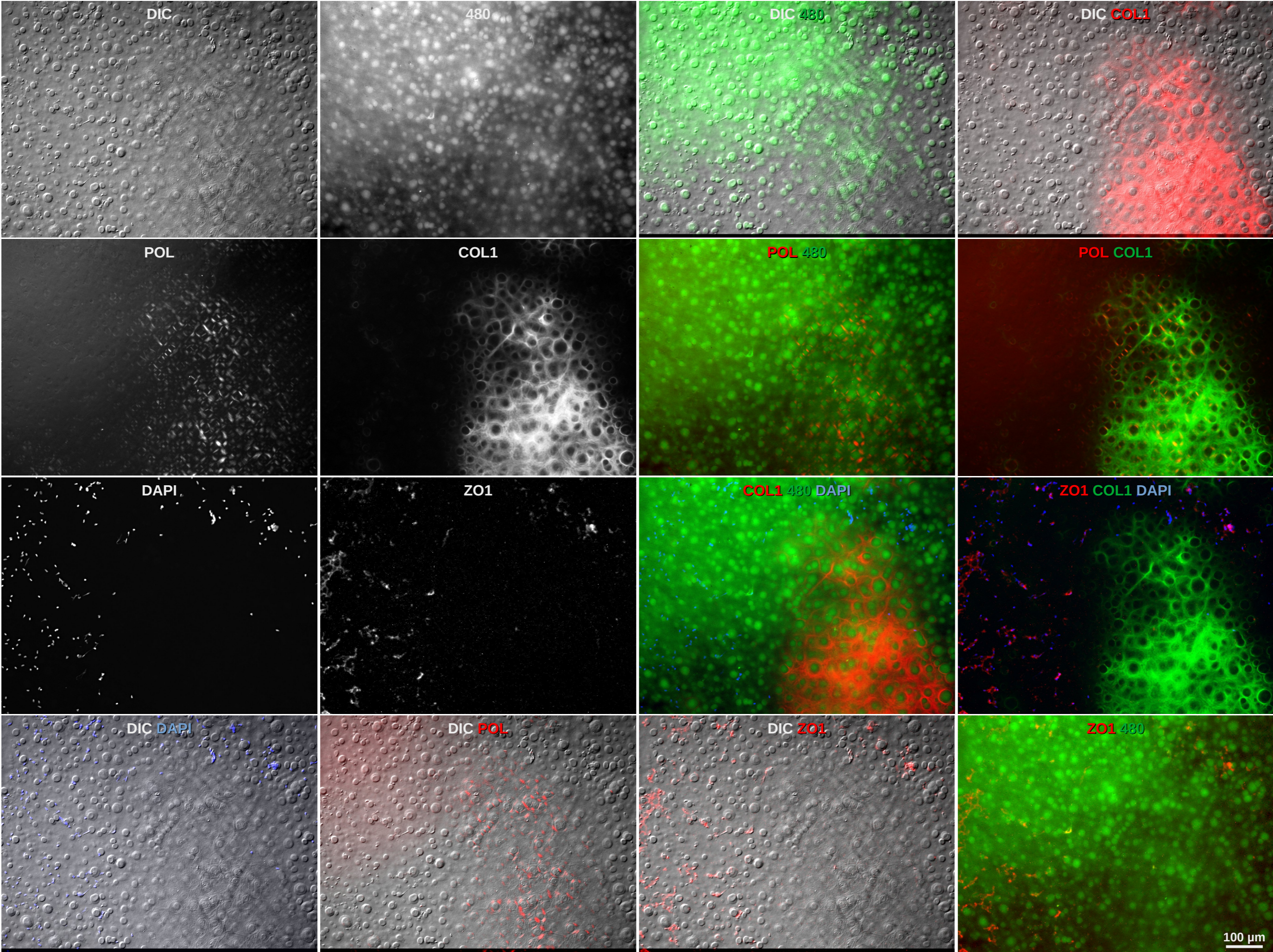

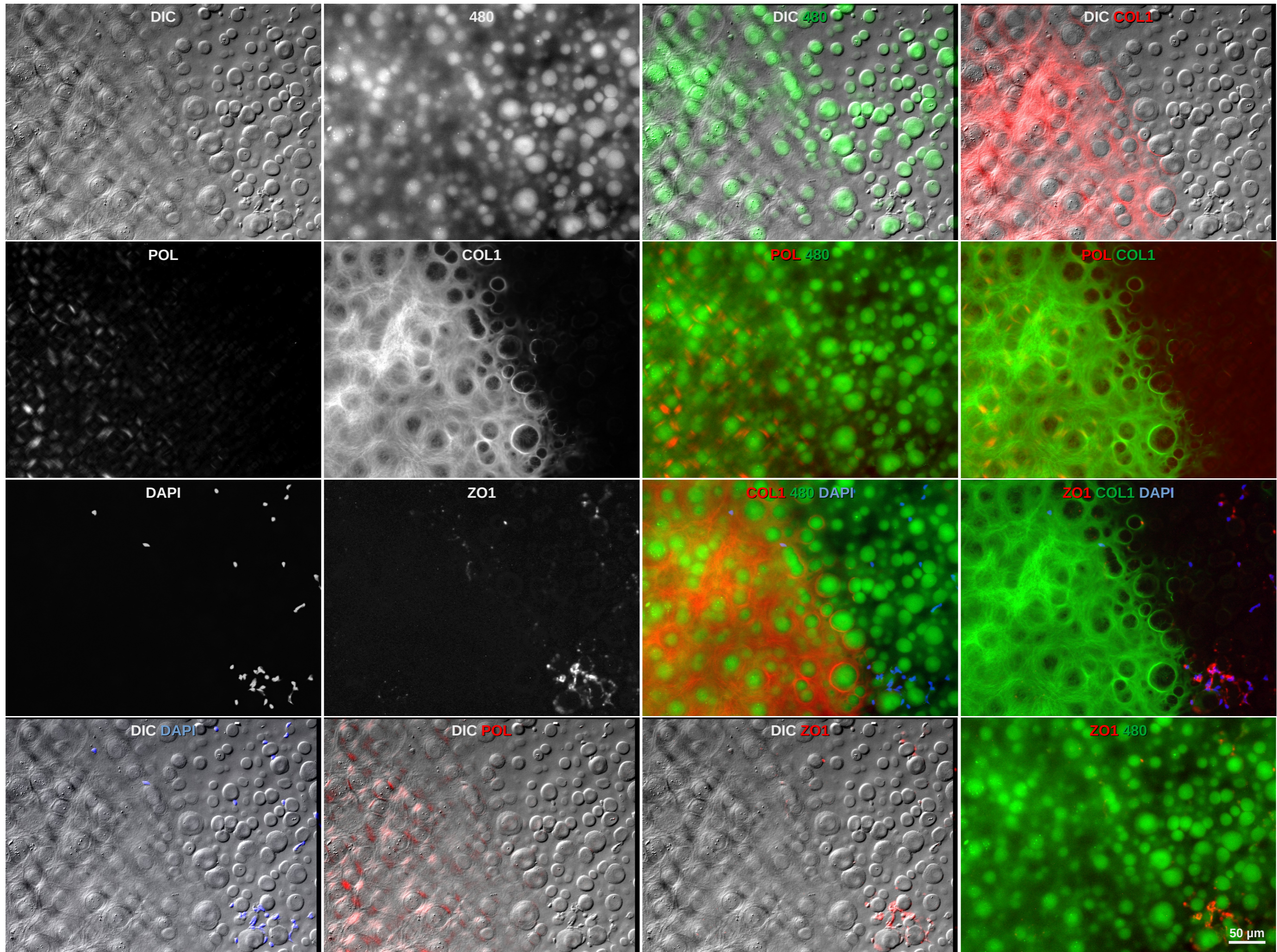

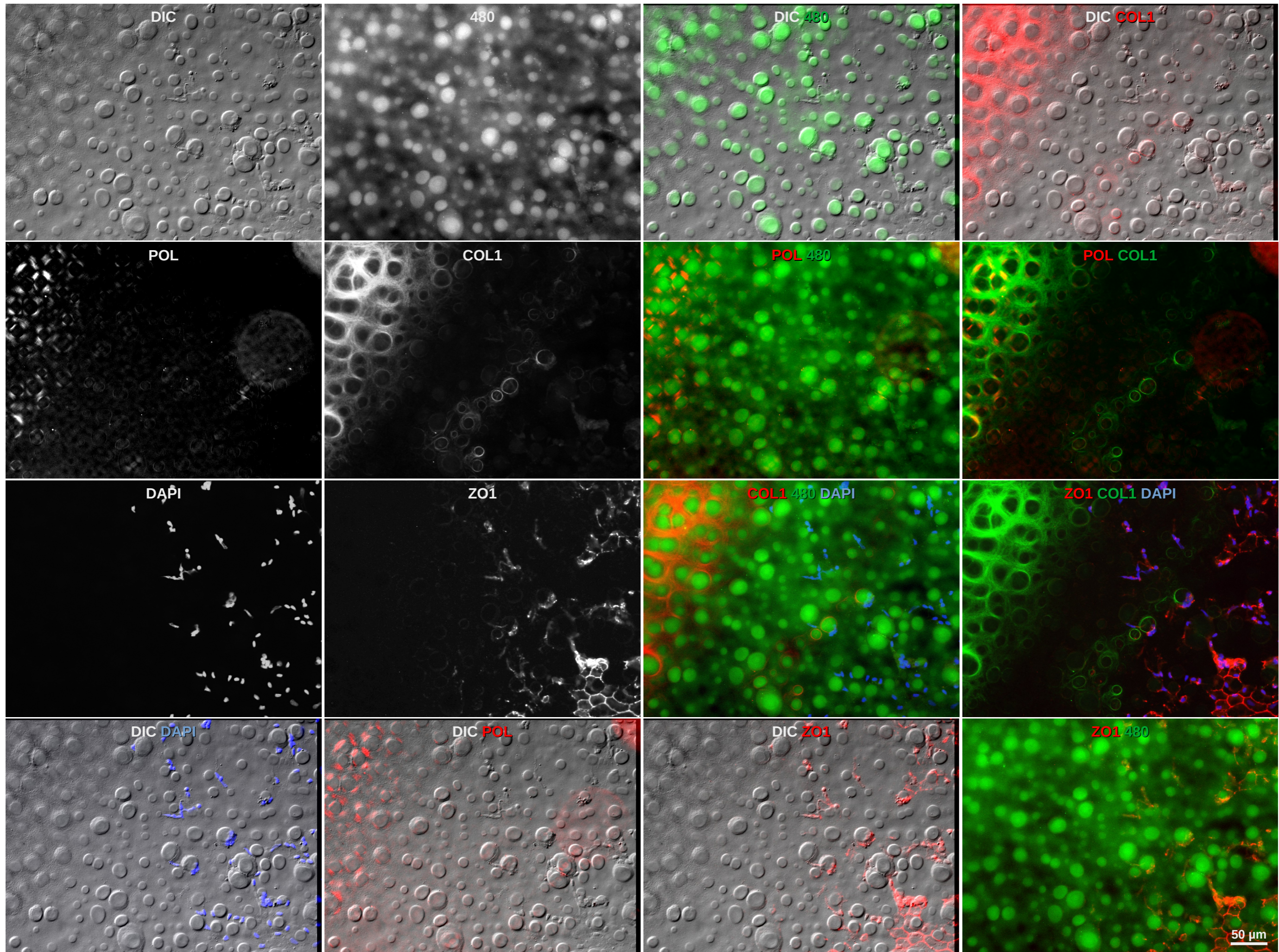

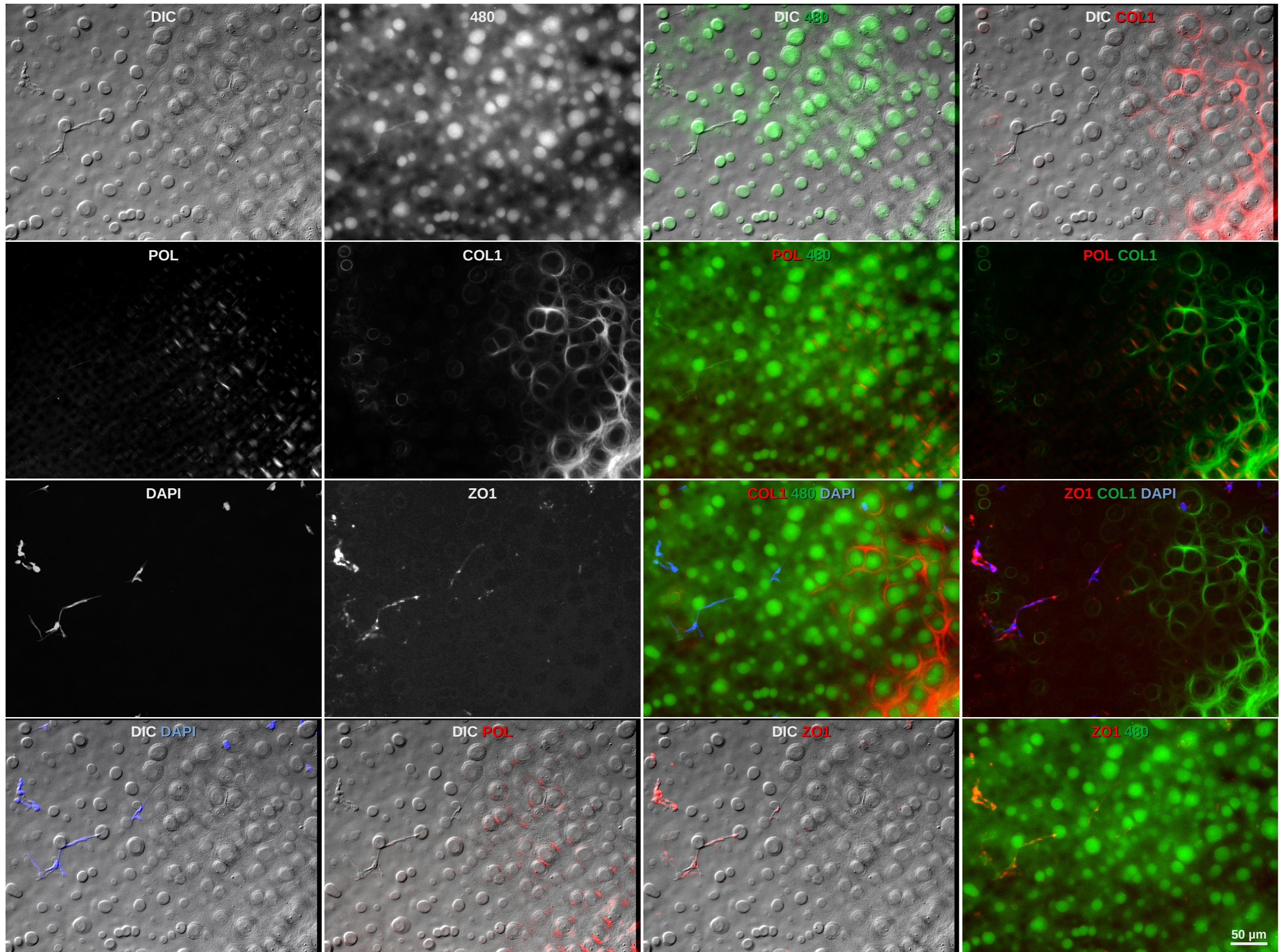

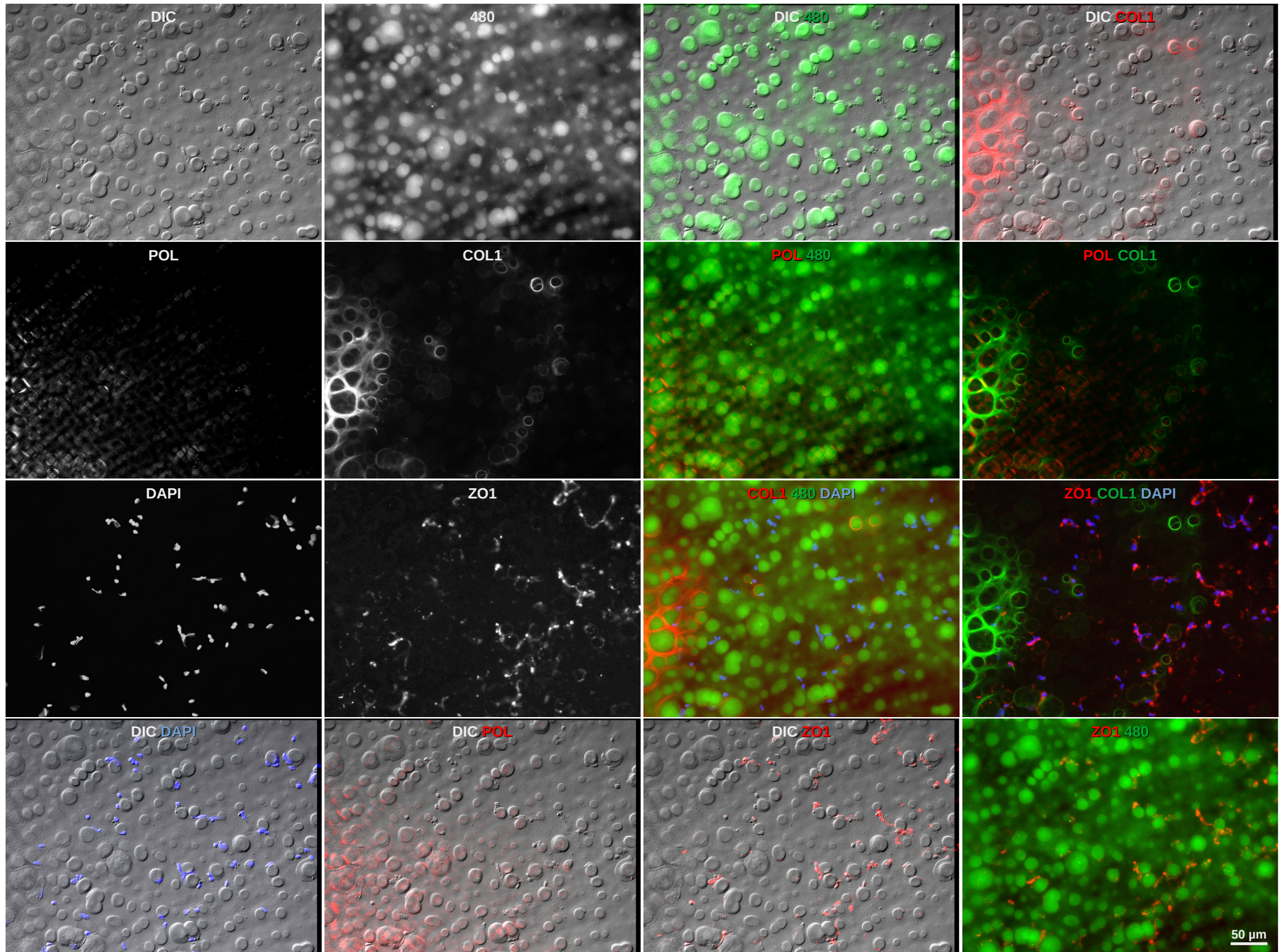

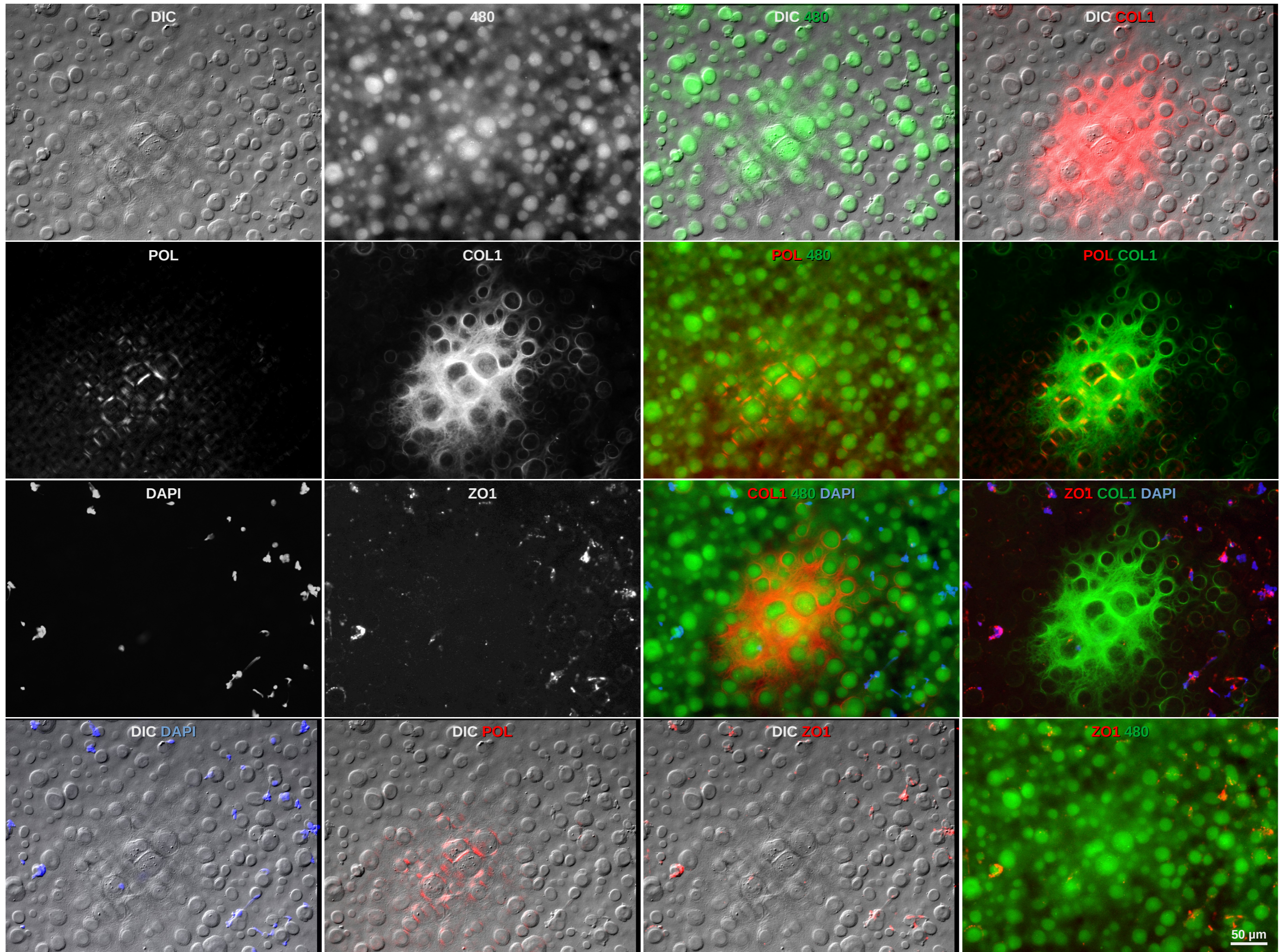

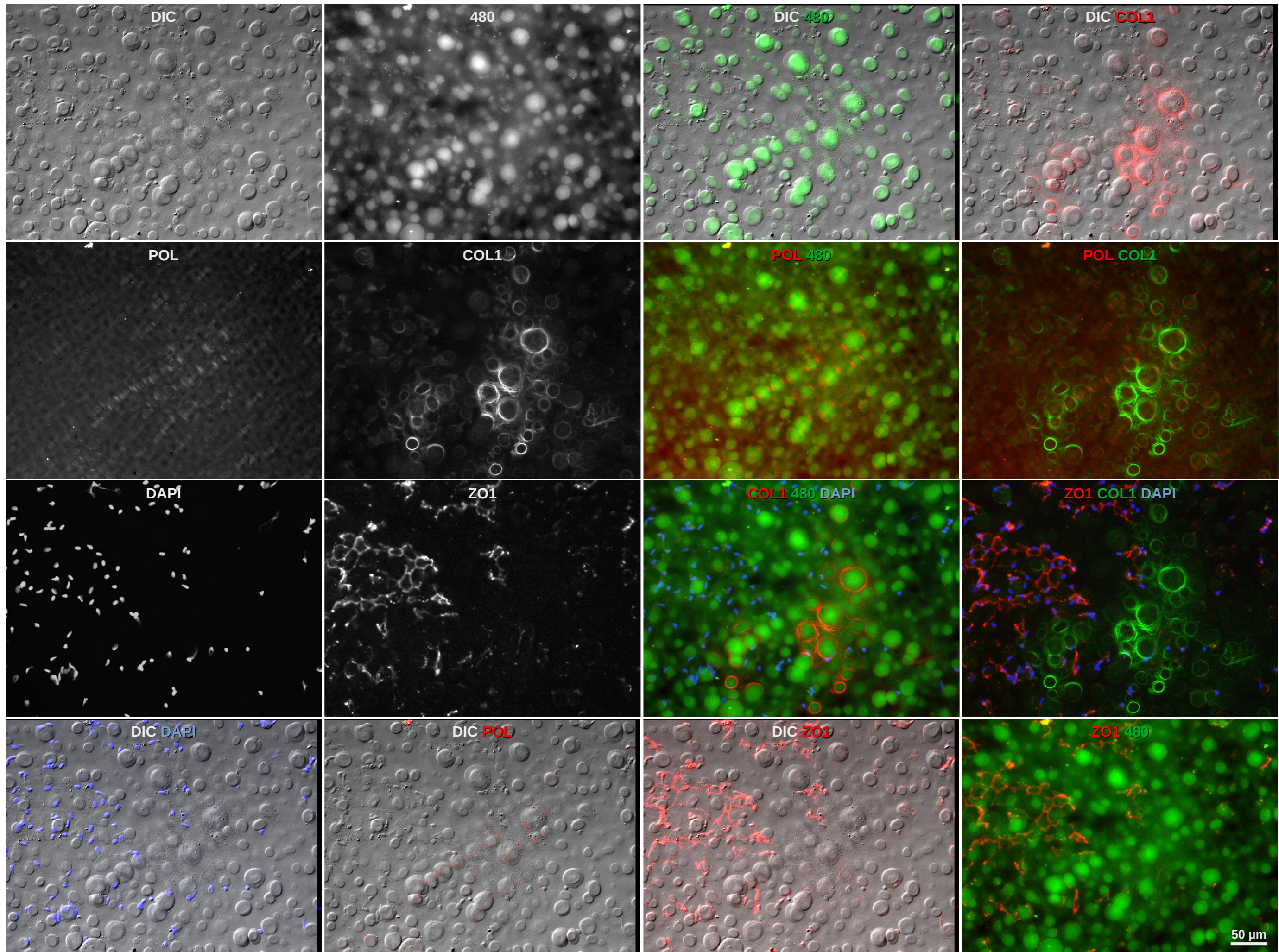

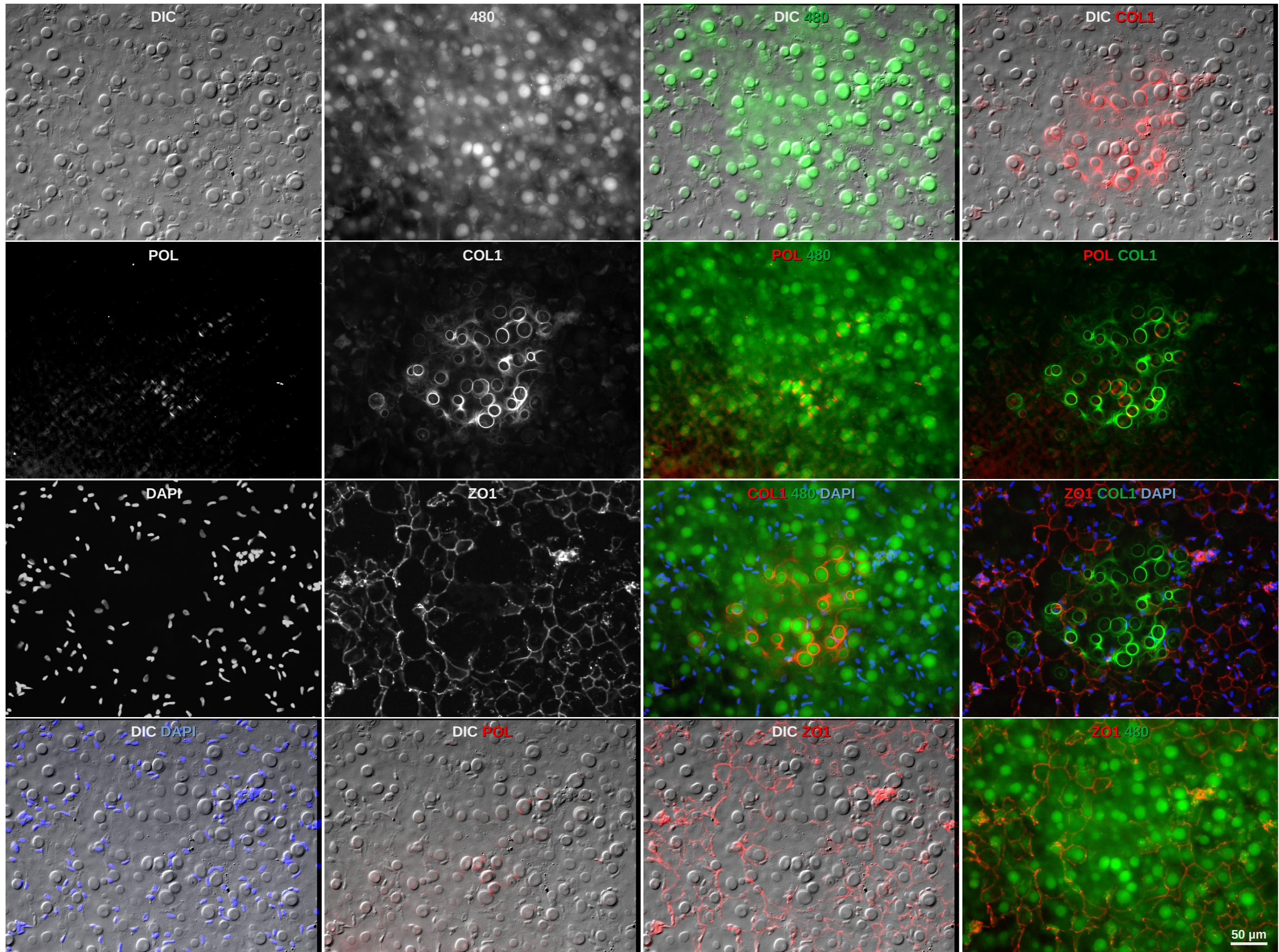

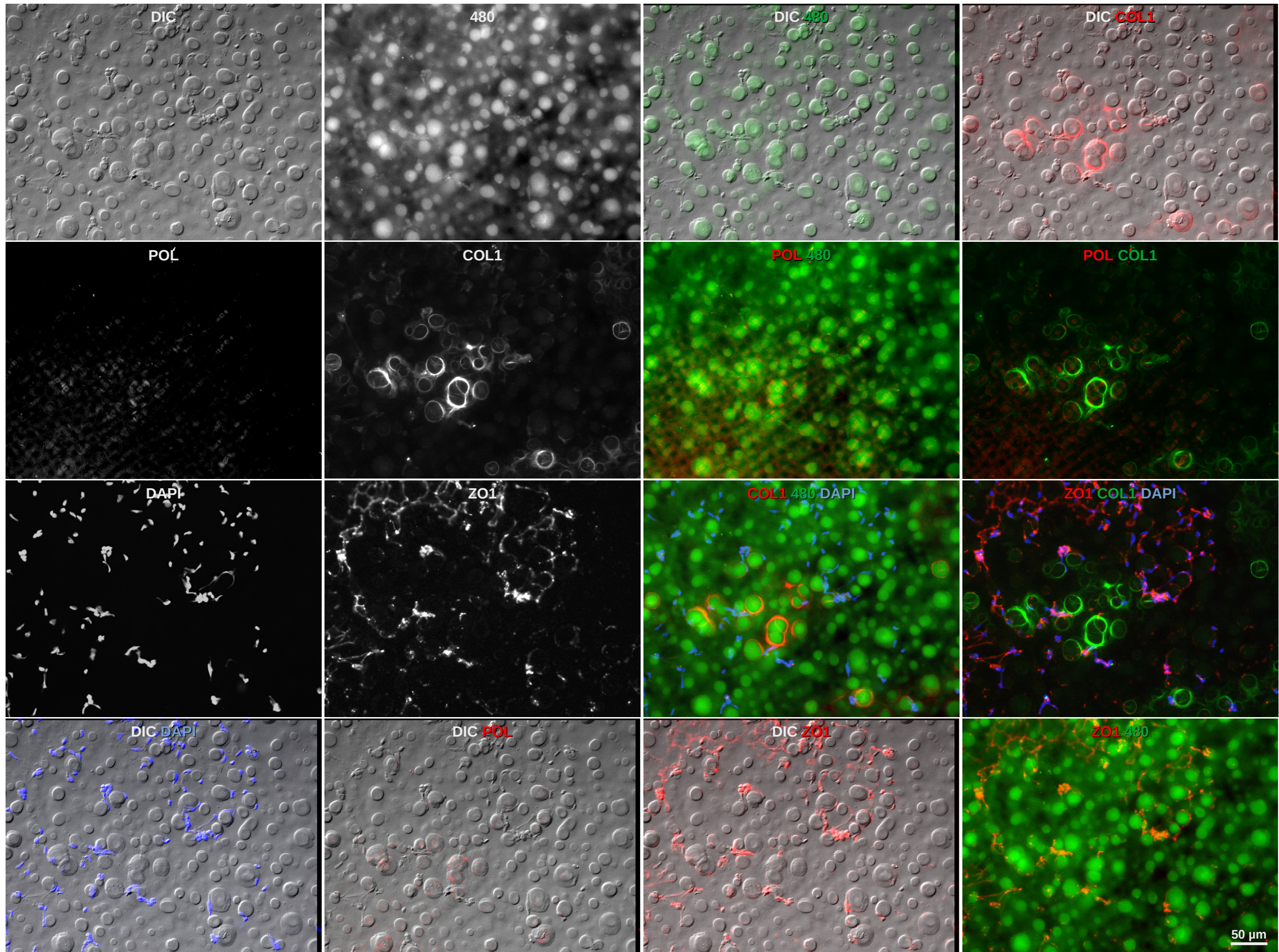

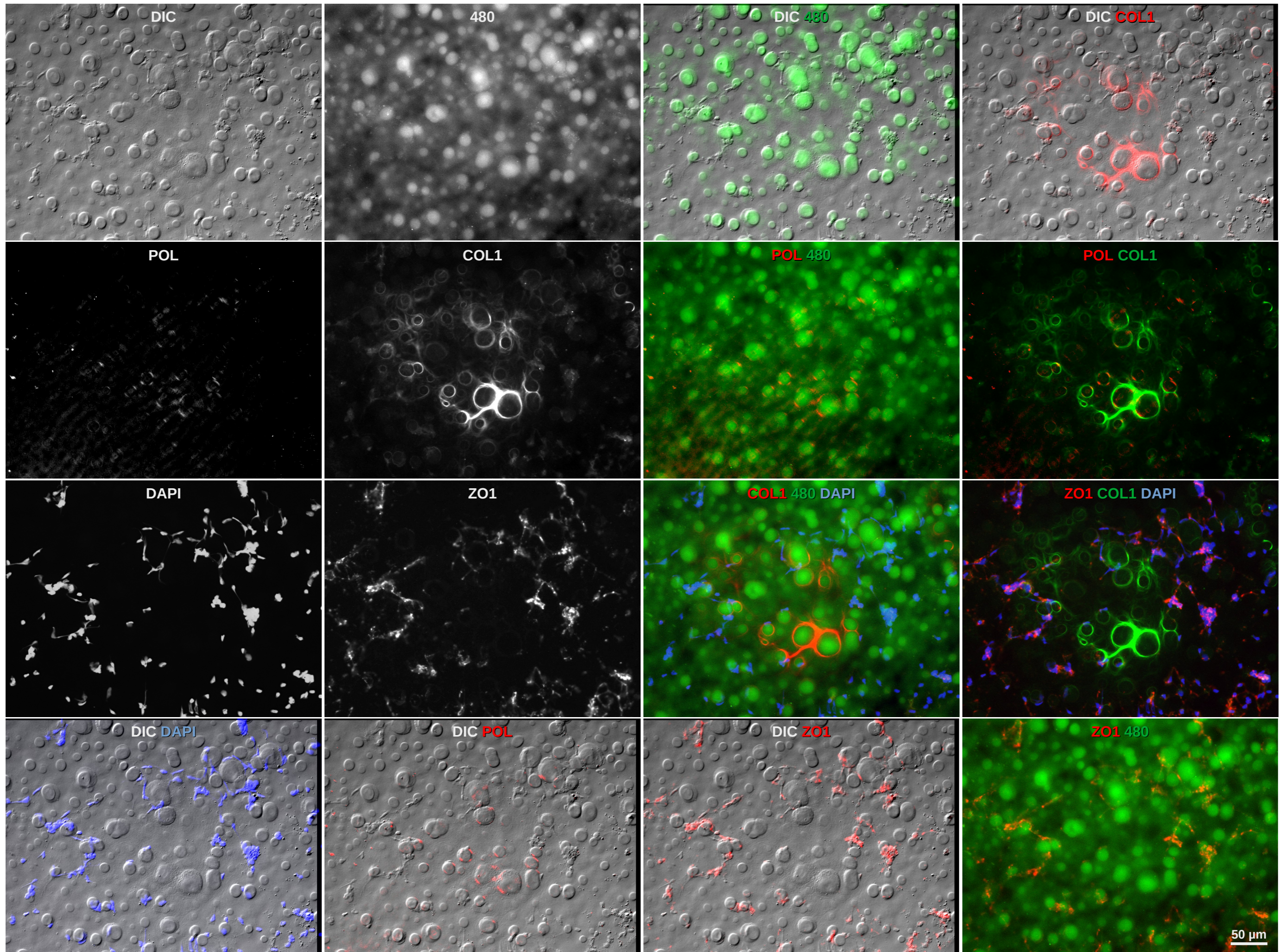

Supplement: Supplementary file 4 — Supplementary Information 4. [file 41598_2026_44926_MOESM4_ESM.pdf]

A1

DIC COL1

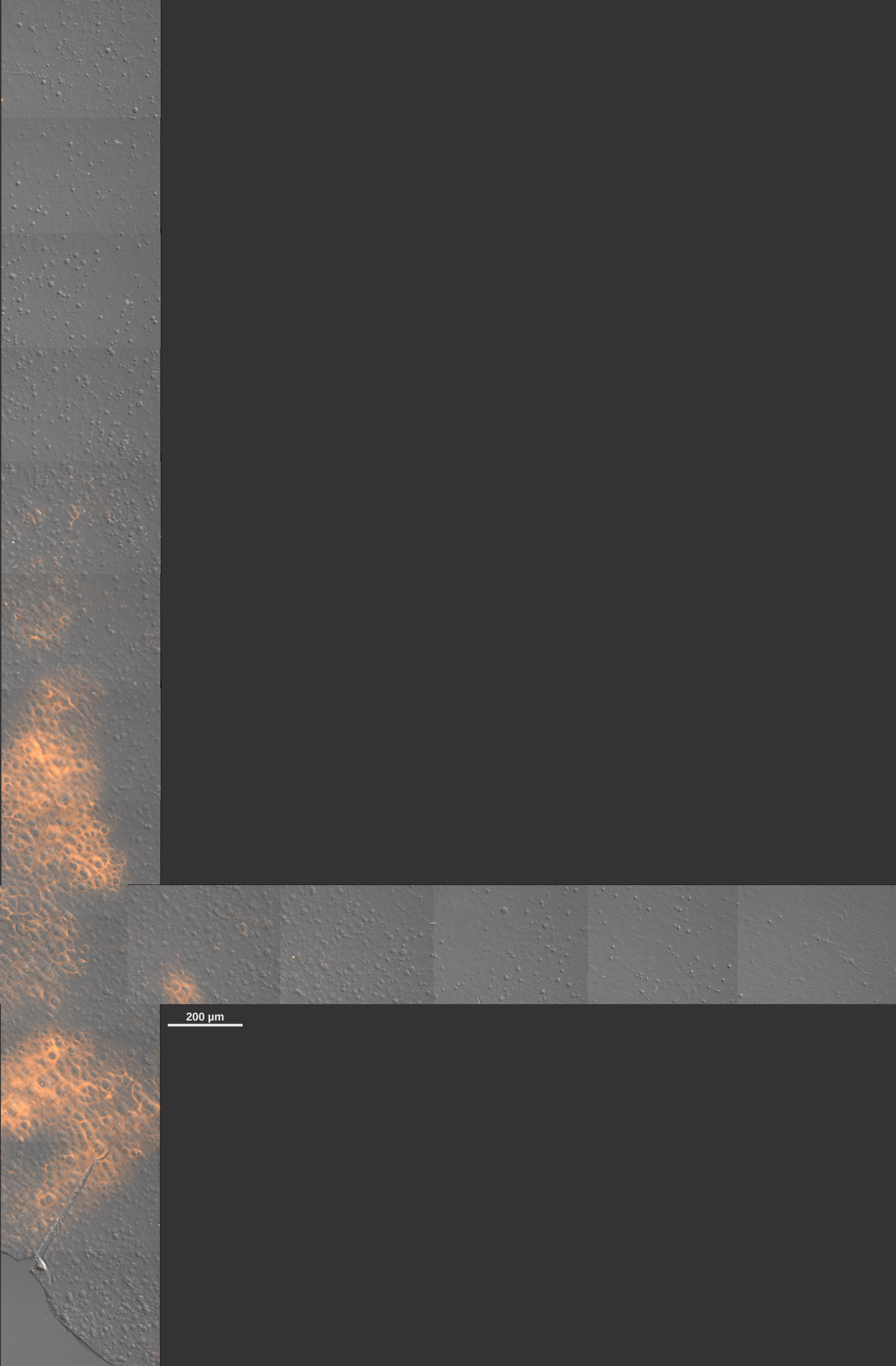

A2

COL1 480 DAPI

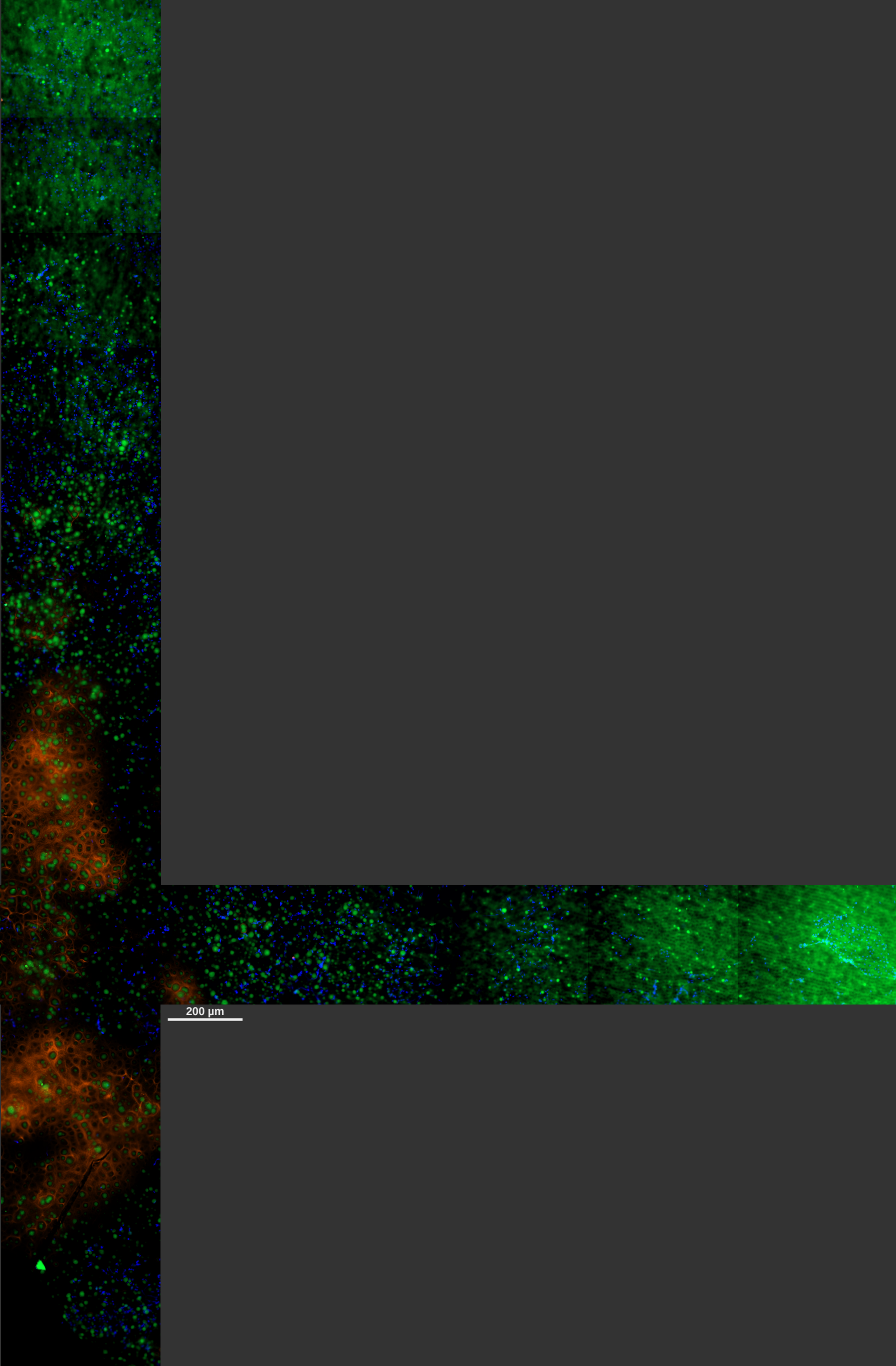

B

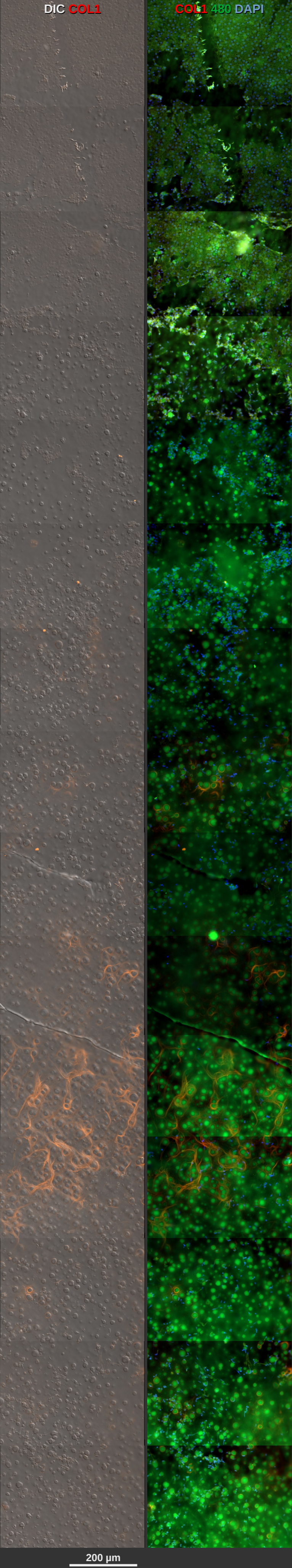

Supplement: Supplementary file 5 — Supplementary Information 5. [file 41598_2026_44926_MOESM5_ESM.pdf]

Suppl. Fig. S6

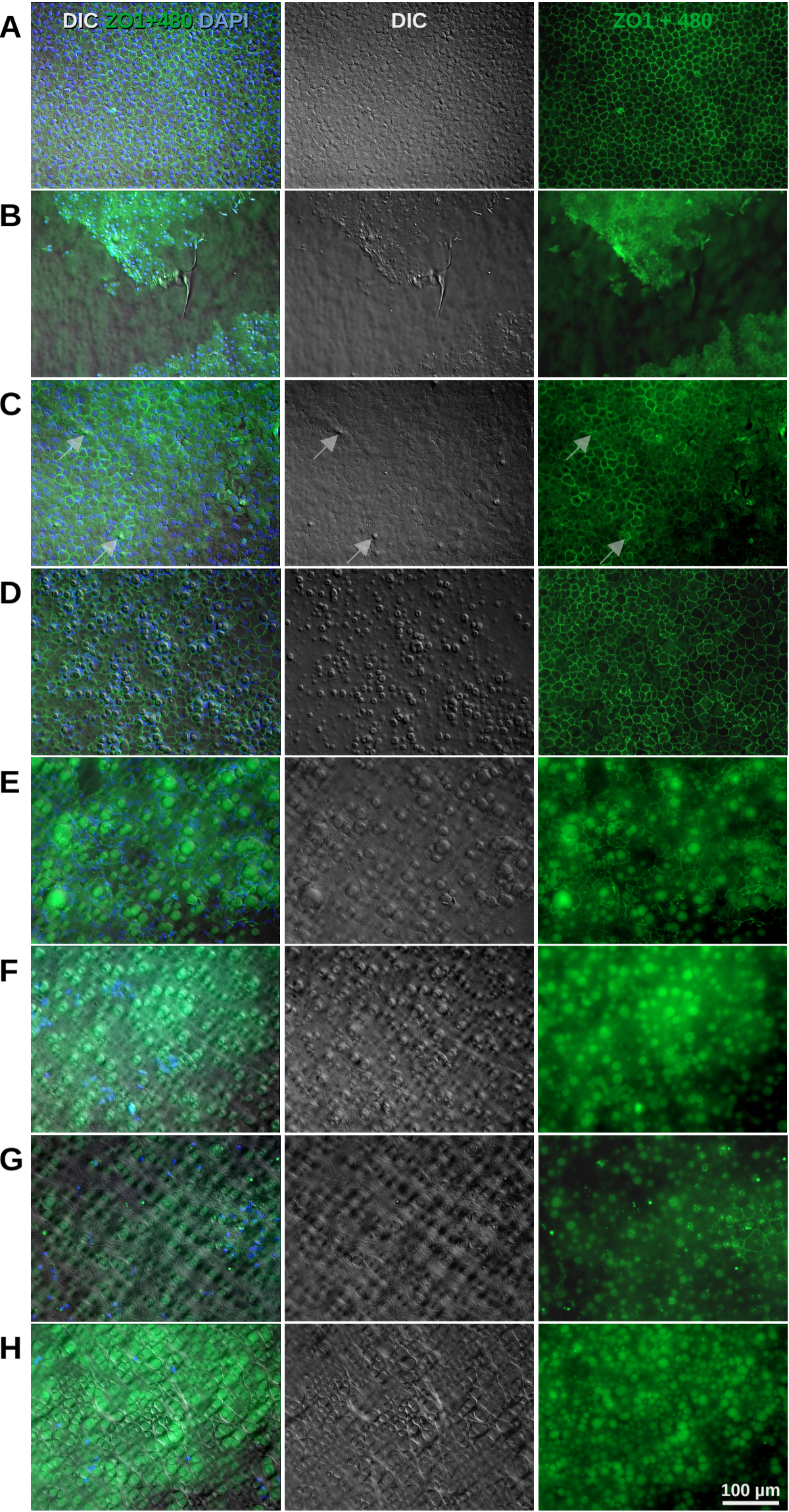

Suppl. Fig. S7

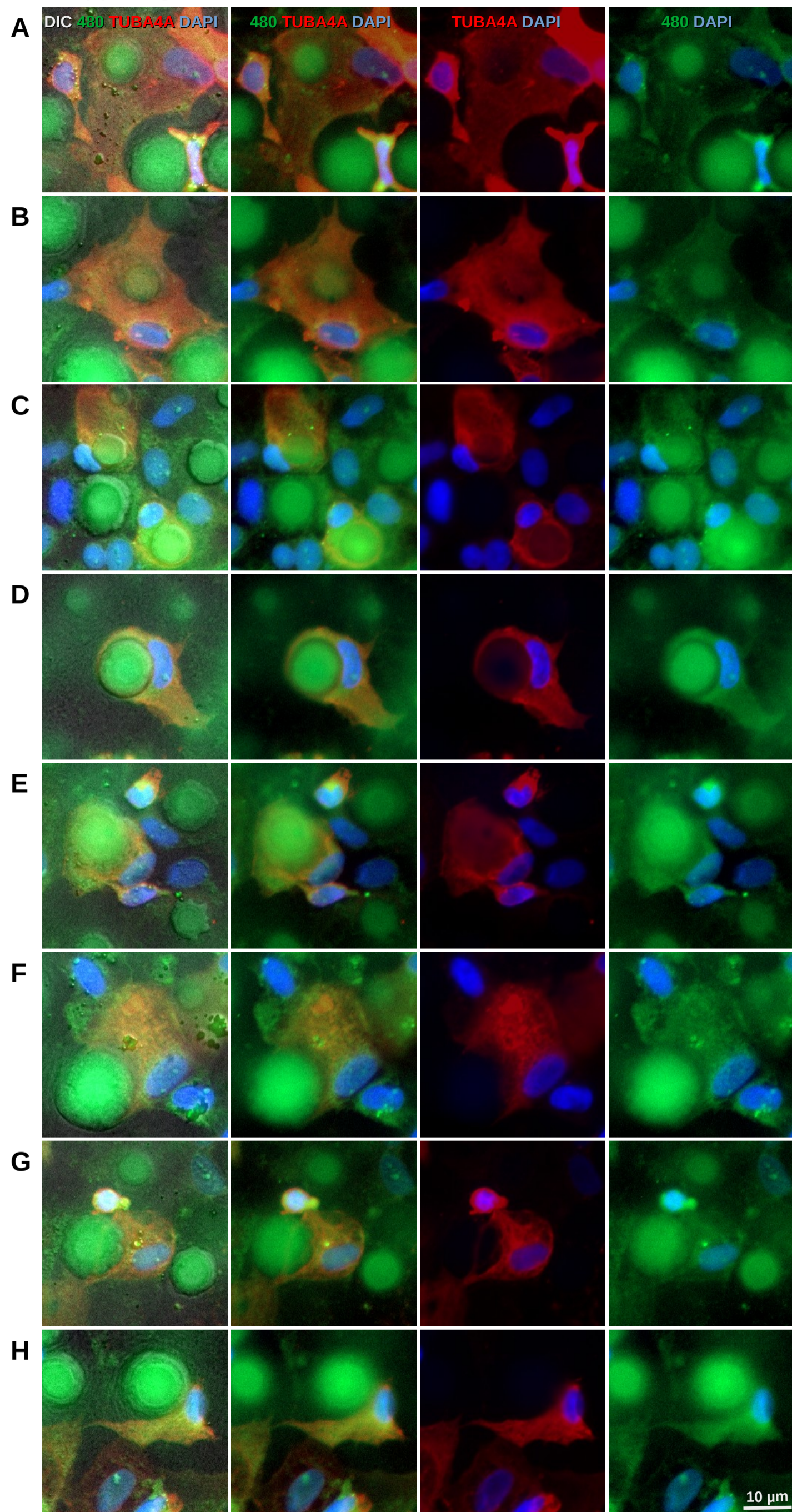

Supplement: Supplementary file 6 — Supplementary Information 6. [file 41598_2026_44926_MOESM6_ESM.pdf]
