# Supplementary material for: Imaging improvements reveal guttae development and posterior fibrillar layer formation in fuchs endothelial corneal dystrophy
Source: Sci Rep. 2026 Mar 26;16:10501. doi: 10.1038/s41598-026-44926-2 (PMC13031840; doi:10.1038/s41598-026-44926-2)
Supplement: Supplementary file 8 — Supplementary Information 7. [file 41598_2026_44926_MOESM8_ESM.docx]

## Suppl. Fig. S1 Peripheral and central guttae in phase contrast microscopy.


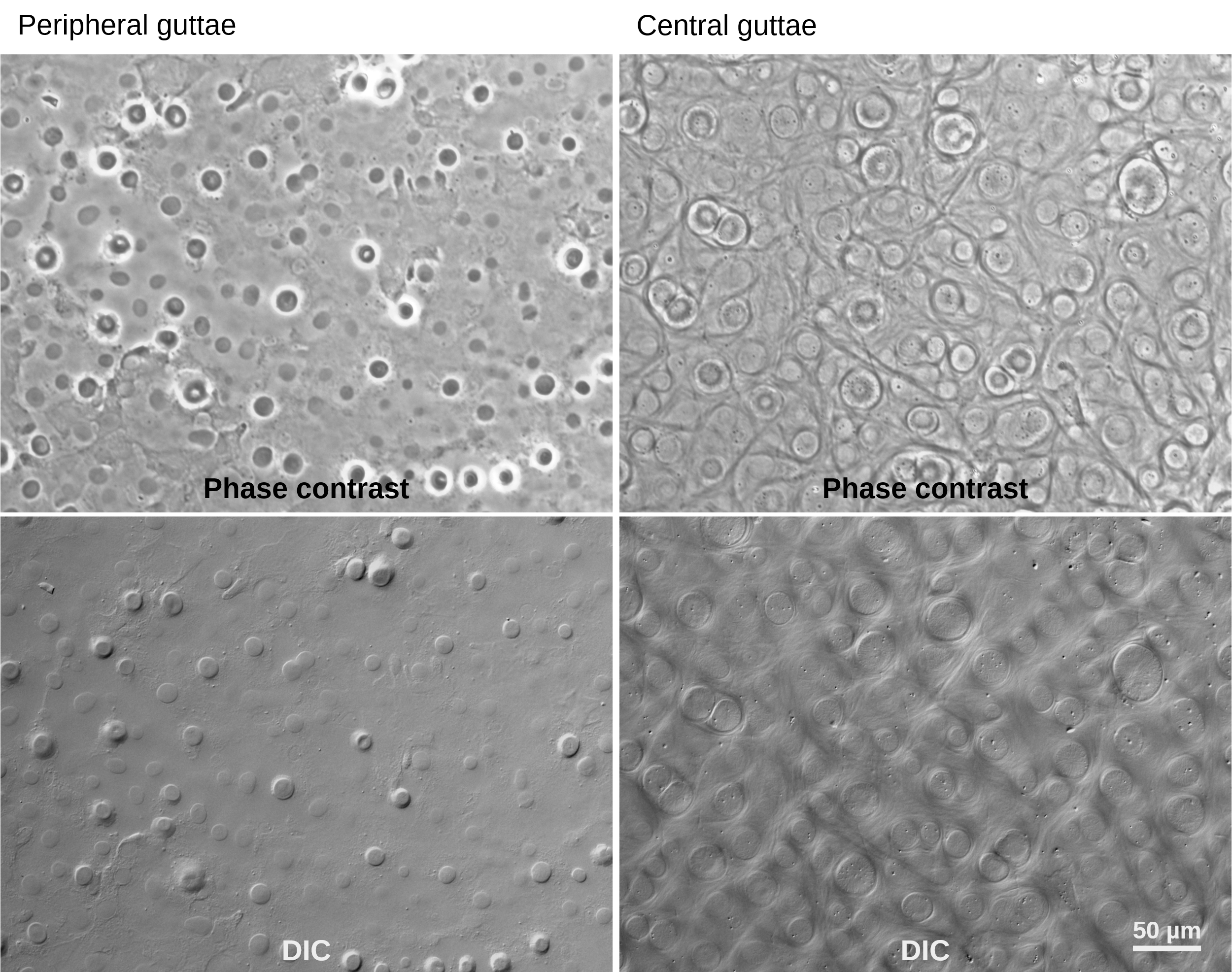


DIC images of a flat-mounted DM show guttae or the PFL in much more detail compared to phase contrast images. The concentric bright rings around peripheral guttae are a typical phase contrast artifact. Note that the DIC images are shifted due to the prisms involved.

## Suppl. Fig. S2 Image series of 9 DM in different stages.

Nine flat-mounted DM (A-I) from mainly late-stage FECD patients, stained for ZO1, are documented by series of adjacent images. Series A shows a rather healthy DM with a few guttae. Series B shows some Hassall-Henle bodies in the first image followed by some images without guttae. When browsing a series, peripheral guttae are followed by central guttae and again by peripheral guttae. A continuous PFL is clearly visible in the central images in most of the series. Although guttae are generally circular, there is some variability in shape, ranging from elliptical to oval. The borders are typically simple and clear, though some more complex patterns are observed. ZO1 staining reveals that guttae predominantly form at the center of CEnCs rather than at the cell borders. The situation becomes more complex in areas where dead cells are replaced by enlarged neighboring cells, resulting in cells that may appear to have multiple nuclei. These supernumerary nuclei are remnants of the previously replaced cells.

## Suppl. Fig. S3 Corneal cross section showing two types of guttae.


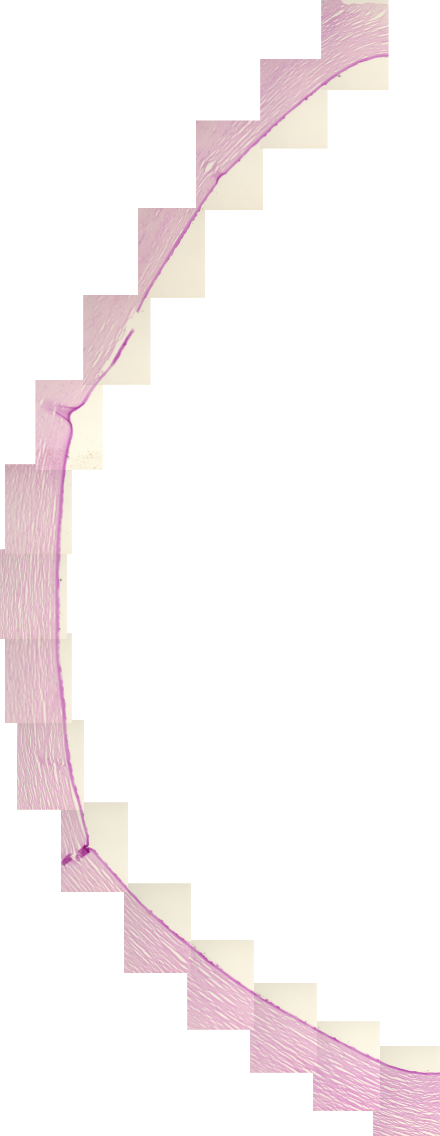


PAS staining of a paraffin section showing peripheral and central guttae. Central guttae were buried within a posterior fibrillar layer while peripheral guttae did not show this layer.

## Suppl. Fig. S4 COL1 staining related to DIC, autofluorescence, PLM, DAPI, and ZO1 staining.

The images of Figs. 4B and 5 were taken in more channels that are presented here.

## Suppl. Fig. S5 Image series showing the distribution of COL1 staining.

Two flat-mounted DM (A and B) were stained for COL1 and combined with DIC, autofluorescence, and nuclei staining. Several areas of COL1 expression were observed, varying in size and intensity. COL1 formed a network surrounding the guttae. Notably, nuclei were rarely found in areas of COL1 expression.

## Suppl. movie 1 Z stack image series of guttae with COL1 staining.

Stack of confocal images of a flat-mounted DM showing a mushroom-like gutta (green) surrounded by a COL1 layer (red). The series begins within the DM and progresses towards the guttae in the CEnC layer. COL1 expression around the two largest guttae in the middle starts with a small ring that enlarges and eventually forms a COL1 network on top of the guttae. The upper part of the two guttae also enlarges, demonstrating a mushroom-like shape. The guttae progressively grow out of the DM without any free-floating parts. Some nuclei are shown in blue. The video may be viewed with a video player from the operating system or with a program like VLC player.

## Suppl. Fig. S6 Different stages of guttae formation.

Flat-mounted DM samples from various FECD patients were stained for ZO1. Representative images illustrate different stages of guttae and posterior fibrillar layer (PFL) development:

1. Normal corneal endothelium exhibited a uniform, regular distribution of cells. Nuclei (also stained blue with DAPI) were visible in DIC and appeared slightly smaller, circular to elliptic, and rougher compared to guttae, which were round and smooth (compare with C).
2. Area of CEnC with circular and elongated nuclei. Parts of the endothelium was mechanically removed so that the surface of the DM is visible.
3. Small guttae began forming beneath the endothelial cell layer (arrows). Most of the nuclei were elongated and the distribution of the nuclei showed a reduced regularity indicating a diseased state of the cells.
4. Peripheral-type guttae were abundant, while endothelial cells were still clearly visible. Fluorescence settings for ZO1 staining minimized the visibility of faint gutta autofluorescence.
   In DIC images, guttae are the dominating structures as compared to cells (compare with B that has larger gaps between areas with cells).
5. Guttae became covered with a thin fibrillar material layer, though endothelial cells and their nuclei remained visible.
6. A thicker PFL formed over guttae blurring the image, with no endothelial cells remaining except for some remnant nuclei.
7. Guttae were entirely covered by a reticular PFL, visible through DIC imaging.
8. Fully developed PFL-covered guttae displayed elevated circular structures atop the PFL. No endothelial cells were present, and only a few nuclei were visible.

## Suppl. Fig. S7 Single CEnCs with guttae.

Single CEnCs with guttae from flat-mounted DM stained for TUBA4A (red) to show the cell body.

1. A cell having a gutta with a small volume and little fluorescent material was surrounded by cells with larger guttae. These cells were much smaller and degenerated and did not cover their gutta anymore (compare with H).
2. A situation similar to A.
3. Two cells having a large gutta filling a large part of the cell. Note that the cytoplasm of the cell was also filled with green fluorescent material.
4. A cell having a large gutta with the cytoplasm moved to one side.
5. A gutta filling most part of the cell.
6. Large gutta with the cell displaced to the side.
7. Large gutta. The cell was moved aside and did not cover the gutta anymore.
8. Degenerating cell alongside a large gutta. Such degenerated cells were quite common.
