# Supplementary material for: Imaging improvements reveal guttae development and posterior fibrillar layer formation in fuchs endothelial corneal dystrophy
Source: Sci Rep. 2026 Mar 26;16:10501. doi: 10.1038/s41598-026-44926-2 (PMC13031840; doi:10.1038/s41598-026-44926-2)

Suppl. Fig. S2

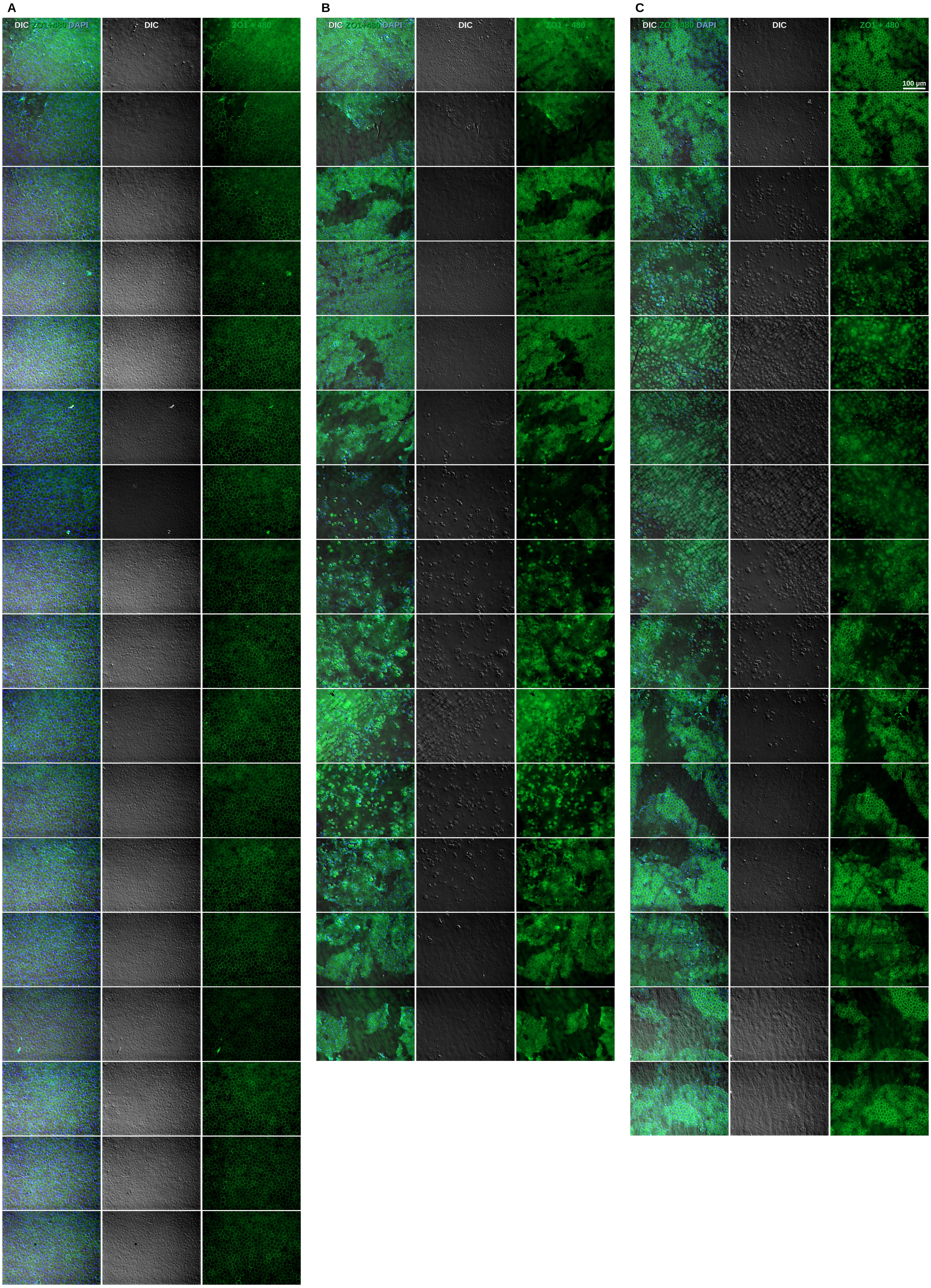

Suppl. Fig. S2

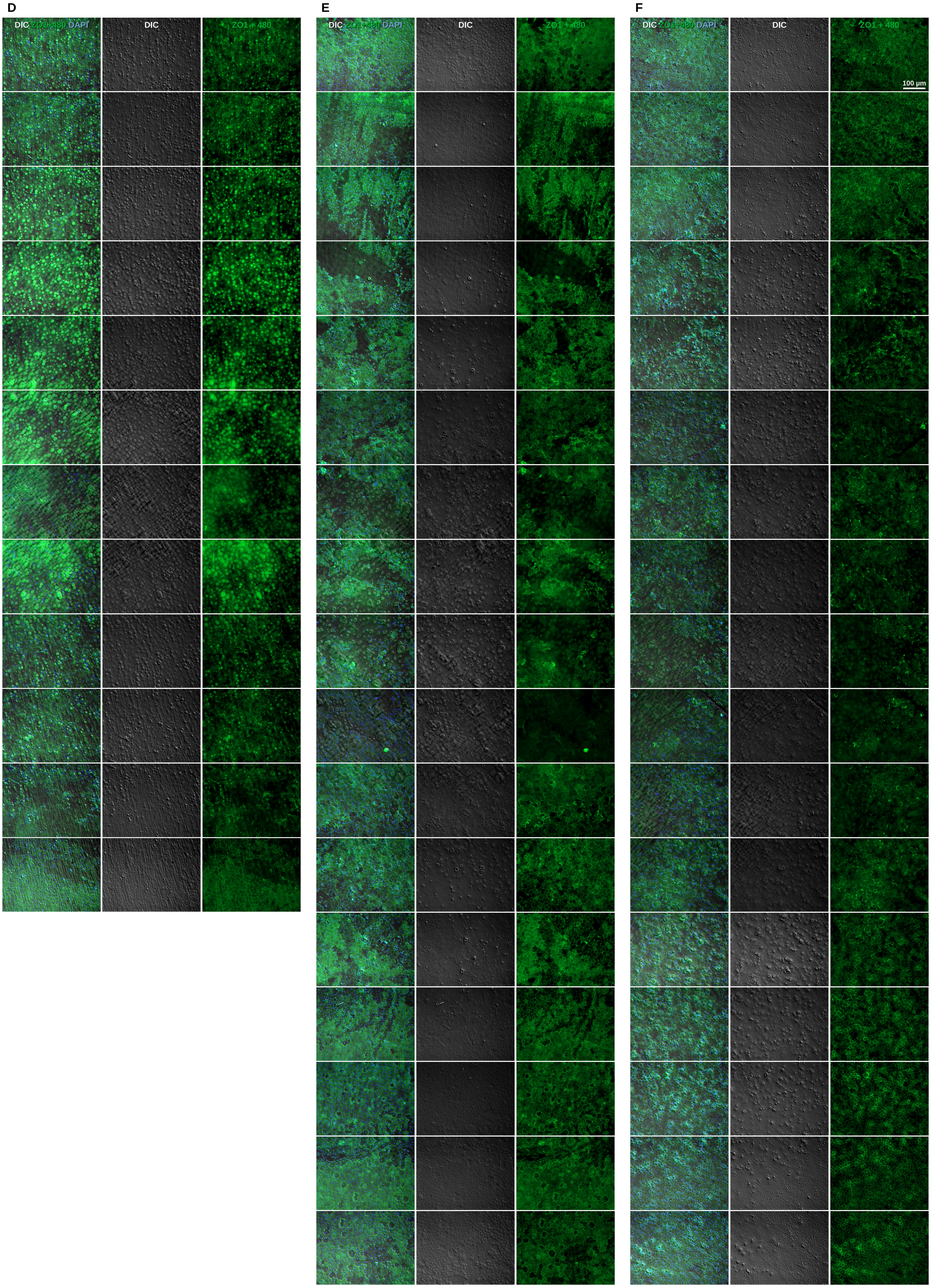
D
DIC ZO1+480 DAPI
DIC
ZO1 + 480
E
DIC ZO1+480 DAPI
DIC
ZO1 + 480
F
DIC ZO1+480 DAPI
DIC
ZO1 + 480
100 µm

Suppl. Fig. S2

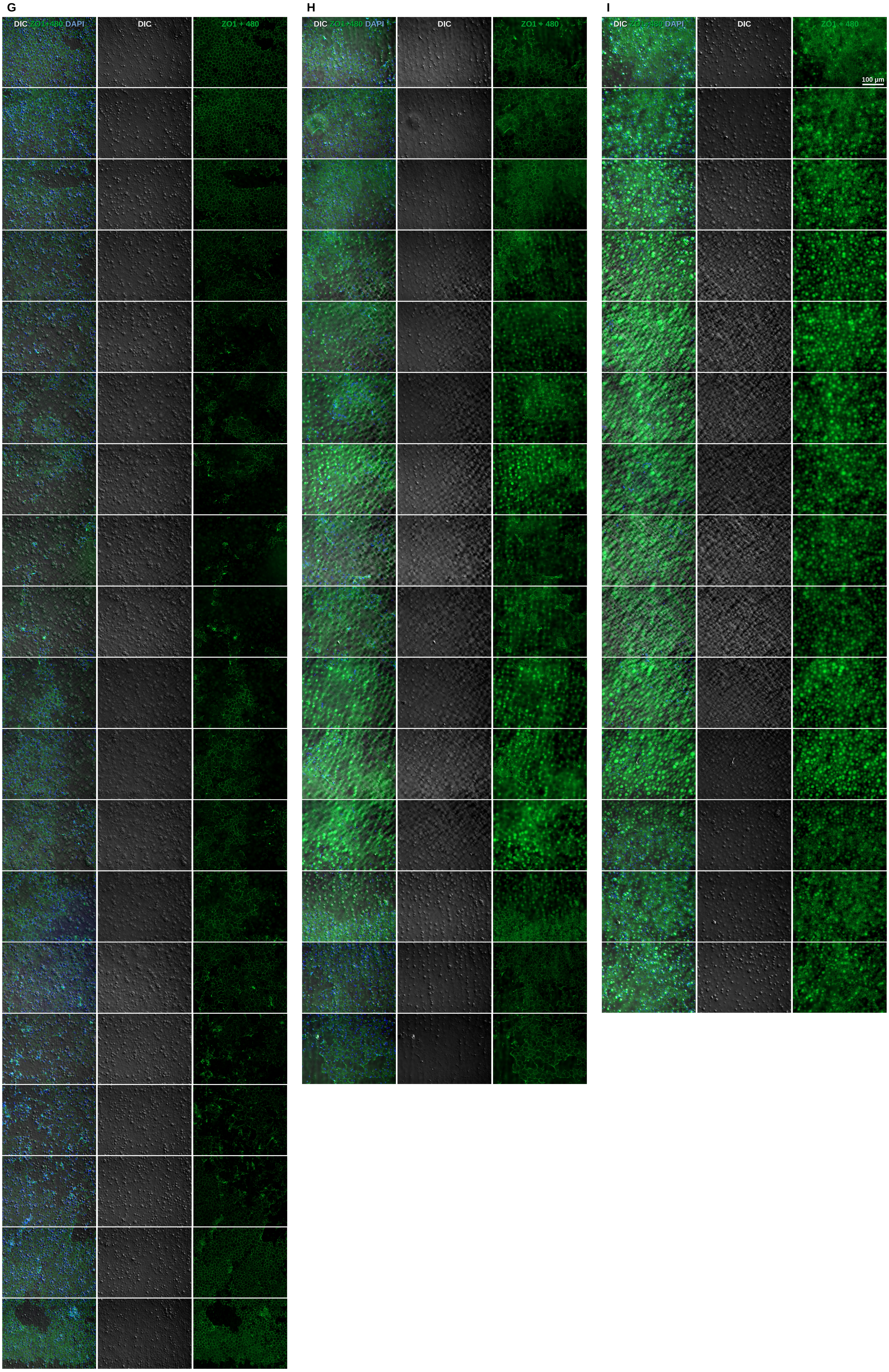

Supplement: Supplementary file 3 — Supplementary Information 3. [file 41598_2026_44926_MOESM3_ESM.pdf]
